# Cross-phenotype genome-wide association study supports shared genetic etiology between skin and gastrointestinal tract diseases

**DOI:** 10.7555/JBR.39.20250166

**Published:** 2026-03-19

**Authors:** Bo Peng, Minghui Jiang, Si Li, Xingyu Chen, Shanshan Cheng, Xingjie Hao

**Affiliations:** 1 Department of Epidemiology and Biostatistics, School of Public Health, Tongji Medical College, Huazhong University of Science and Technology, Wuhan, Hubei 430030, China; 2 Ministry of Education Key Laboratory of Environment and Health, School of Public Health, Tongji Medical College, Huazhong University of Science and Technology, Wuhan, Hubei 430030, China; 3 Department of Neurology, Beijing Tiantan Hospital, Capital Medical University, Beijing 100070, China; 4 China National Clinical Research Center for Neurological Diseases, Beijing 100070, China

**Keywords:** gut-skin axis, gastrointestinal tract diseases, skin diseases, pleiotropic analysis, Mendelian randomization

## Abstract

The comorbidity of skin and gastrointestinal tract (GIT) diseases, primarily driven by the gut-skin axis (GSA), is well established. However, the genetic contribution to the GSA remains unclear. Here, using genome-wide association study (GWAS) summary statistics from European populations, we performed a genome-wide pleiotropic analysis to investigate the shared genetic basis and causal associations between skin and GIT diseases. We observed extensive genetic correlations and overlaps between skin and GIT diseases. A total of 298 pleiotropic loci were identified, 75 of which were colocalized, and 61 exhibited pleiotropic effects across multiple trait pairs, including 2p16.1 (*PUS10*), 6p21.32 (*HLA-DRB1*), 10q21.2 (*ZNF365*), and 19q13.11 (*SLC7A10*). Additionally, five novel loci were identified based on the pleiotropic analysis; among them, the 15q22.2 locus harboring *RORA* was validated by the latest inflammatory bowel disease GWAS. Gene-based analysis identified 394 unique pleiotropic genes, which were enriched in GSA-associated tissues and the immune system, and protein-protein interaction analysis further revealed that the GPCR-cAMP, chromatin remodeling, JAK-STAT, and HLA-mediated immunity pathways were involved in GSA comorbidity. Notably, the JAK-STAT pathway showed strong potential for drug repurposing, with adalimumab targeting tumor necrosis factor and ustekinumab targeting interleukin-12 subunit beta already being used to treat both skin and GIT diseases. Finally, Mendelian randomization analysis identified five significant causal associations, and subsequent mediation analysis identified three potential microbiota-GIT-skin pathways. Taken together, our study demonstrated that the shared genetic factors between skin and GIT diseases were widely distributed across the genome. These findings will enhance our understanding of the genetic mechanisms underlying GSA comorbidity.

## Introduction

The comorbidity of skin and gastrointestinal tract (GIT) diseases has been well recognized^[1]^. For instance, Kim *et al*^[2]^ reported that patients with inflammatory bowel disease (IBD) were at higher risk of skin diseases, including atopic dermatitis (AD), psoriasis, and rosacea. Similarly, Piontkowski *et al*^[3]^ found that rosacea patients faced increased risks of gastro-oesophageal reflux disease (GORD), Crohn's disease, ulcerative colitis, and irritable bowel syndrome (IBS). This comorbidity is mainly regulated by the gut-skin axis (GSA), a bidirectional relationship between skin and GIT that links skin disorders with intestinal dysfunction and inflammation^[1,4]^. Both environmental factors, such as gut microbiota^[5]^, and genetic factors participate in the GSA. However, the extent to which genetics influence the GSA is still poorly understood.

The release of large-scale genome-wide association study (GWAS) summary data^[6–7]^ and omics data^[8]^ enables exploration of the genetic etiology of skin and GIT diseases. Several shared loci have been identified, such as *C11orf30* for AD and IBD^[9]^, *IRF5* and *PRDM1* for systemic lupus erythematosus (SLE) and IBD^[10]^, as well as *NOD2*/*CARD15* for hidradenitis suppurativa (HS) and IBD^[11]^. However, these studies only focused on loci that were significant in both IBD and skin disease GWASs. Cross-trait analysis offers a means to elucidate genetic etiology between multiple diseases by leveraging shared pleiotropy^[12]^. While previous studies have revealed the shared genetic basis of the gut-brain axis^[13]^, the gut-lung axis^[14]^, and the heart-brain axis^[15]^, the GSA remains unexplored. Further investigation into the genetic etiology between skin and GIT diseases not only enhances our understanding of GSA interactions but also holds potential for drug repurposing and simultaneous treatment.

**Figure 1:**
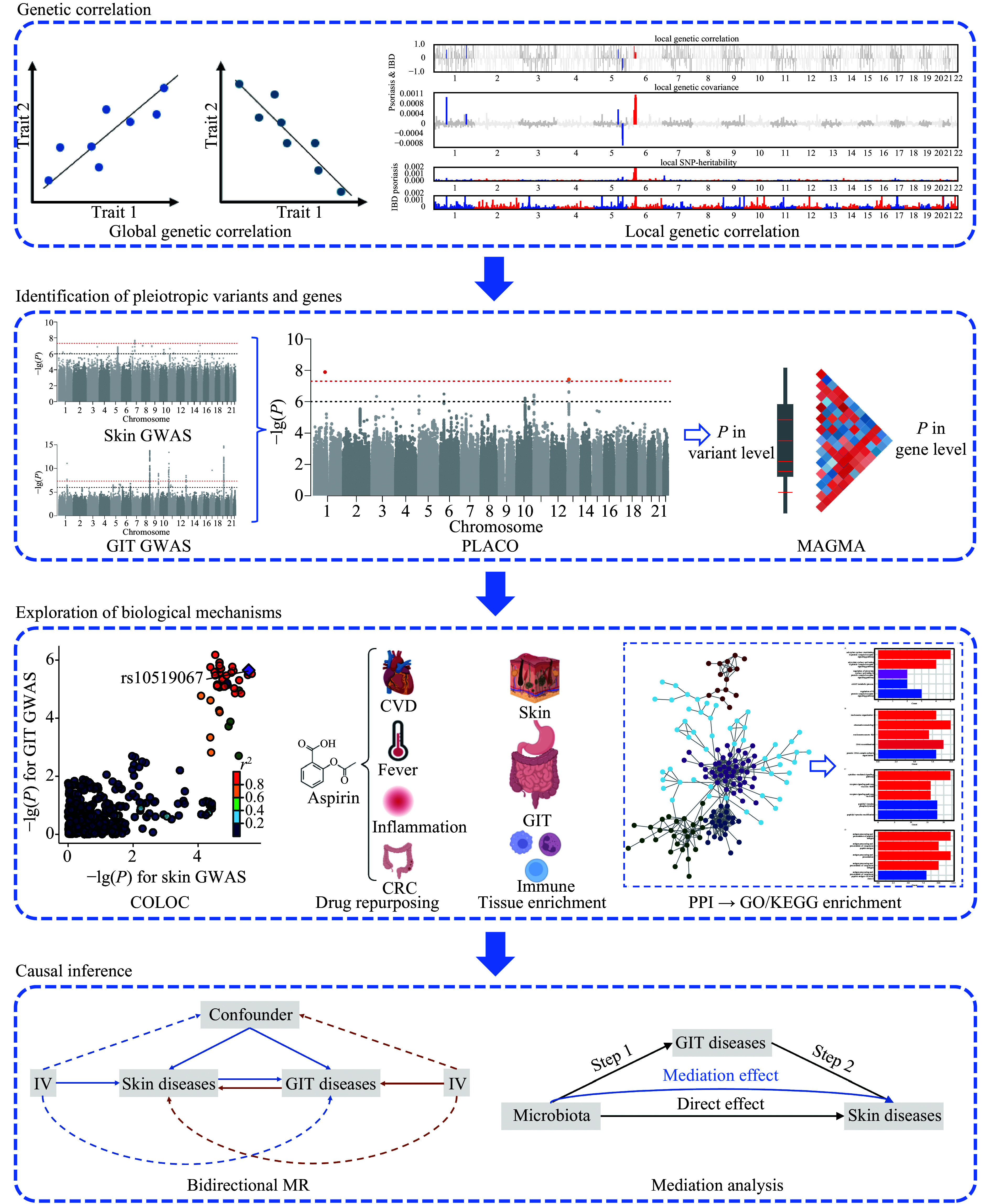
Analysis workflow. First, genetic correlations were evaluated at both global and local scales. Second, pleiotropic variants and genes were identified using PLACO and MAGMA. Third, colocalization, drug repurposing, tissue enrichment, PPI, and subsequent GO/KEGG enrichment analyses were conducted to explore biological mechanisms. Bidirectional MR and mediation analyses were performed to infer causal relationships and assess the potential role of microbiota between skin and GIT diseases. Abbreviations: CVD, cardiovascular disease; CRC, colorectal cancer; GIT, gastrointestinal tract; GO, gene ontology; GWAS, genome-wide association study; IV, instrumental variable; KEGG, Kyoto Encyclopedia of Genes and Genomes; MAGMA, multimarker analysis of genomic annotation; MR, Mendelian randomization; PLACO, pleiotropic analysis under composite null hypothesis; PPI, protein-protein interaction.

To this end, we conducted a genome-wide pleiotropic analysis between skin and GIT diseases. The workflow is shown in ***Fig. 1***. Specifically, for genetic correlation, we used linkage disequilibrium score regression (LDSC)^[16]^ and ρ-HESS^[17]^ to identify global and local genetic correlations, respectively. Subsequently, for genetic overlap, we explored the genetic pleiotropy at the variant, gene, and pathway levels. Additionally, bidirectional Mendelian randomization (MR) and mediation analyses were performed to infer causal relationships and the potential role of microbiota between skin and GIT diseases.

## Materials and methods

### GWAS summary data

We obtained summary statistics for seven skin diseases, including acne (34422 cases and 364991 controls), AD (15208 cases and 367046 controls), HS (1070 cases and 394105 controls), psoriasis (10312 cases and 397564 controls), rosacea (2455 cases and 394105 controls), SLE (5201 cases and 9066 controls), and urticaria (11187 cases and 398204 controls), as well as for four GIT diseases, including peptic ulcer disease (PUD, 16666 cases and 439661 controls), GORD (54854 cases and 401473 controls), IBD (25042 cases and 34915 controls), and IBS (53400 cases and 433201 controls). All GWASs were conducted in European populations, with details provided in ***Supplementary Table 1***.

### Genetic correlation estimation

We used LDSC^[16]^ to estimate the global genetic correlation for 28 trait pairs. Given that LDSC lacks precise quantification of the local genomic regions and may be influenced by unbalanced local genetic covariance, we further quantified local genetic correlation using ρ-HESS^[17]^. In ρ-HESS, the genome was partitioned into 1703 independent linkage disequilibrium (LD) blocks^[17]^, with significance of each block declared at 2.94 × 10^−5^ (0.05/1703). These local genetic correlations were then aggregated to derive the global genetic correlation.

### Identification of pleiotropic loci

Pleiotropic variants were identified using pleiotropic analysis under a composite null hypothesis (PLACO)^[12]^. The PLACO statistic is defined as $ {T}_{PLACO}={Z}_{1}{Z}_{2} $, with the null hypothesis being $ {\beta }_{1}{\beta }_{2}=0 $^[12]^. Significant pleiotropic variants were identified if *P*_*PLACO*_ < 5 × 10^−8^. Clumping was performed using PLINK^[18]^ with LD *r*^2^ < 0.1 in 500 kb based on 1000 Genomes Project Phase 3 Europeans^[19]^. Loci less than 250 kb apart were further merged using BEDTools^[20]^. The lead variant of each locus was annotated using ANNOVAR^[21]^. A novel locus was defined if the lead variant was located more than 500 kb from the significant variants in the original GWAS or the NHGRI-EBI GWAS catalog^[22]^ (as of May 1, 2024).

### Colocalization analysis

A 100 kb region around each lead variant was selected to determine whether a causal variant was shared between pairwise skin and GIT diseases using coloc^[23]^. Five hypotheses were tested for each region, H_0_: associated with neither disease; H_1_ or H_2_: only associated with a skin disease or a GIT disease; H_3_: associated with two diseases but with different causal variants; H_4_: associated with two diseases and with the same causal variant. Default arguments were used, with a prior probability of 1 × 10^−4^ for H_1_ or H_2_ and 1 × 10^−5^ for H_4_. A region was declared colocalized if the posterior probability of H_4_ (PP4) exceeded 0.7^[13–14]^.

### Identification of pleiotropic genes

Multimarker analysis of GenoMic annotation (MAGMA)^[24]^ was performed on PLACO results using the FUMA platform^[25]^ to identify pleiotropic genes. The pleiotropic *P*-values of variants at each gene were integrated into the gene level *P*_MAGMA_ based on the SNP-wide mean model and the 1000 Genomes Phase 3 European panel^[19]^. The significance of pleiotropic genes was declared at *P*_MAGMA_ < 2.72 × 10^−6^ (0.05/18354).

### Drug repurposing analysis

DrugBank^[26]^ is a comprehensive drug database that offers information on drug-drug and drug-gene interactions. The latest version, 5.1.12 (downloaded on May 27, 2024), contains over 16000 drugs, of which more than 10000 are approved or investigational. We queried this database to identify drugs targeting pleiotropic genes, considering only approved or investigational drugs.

### Tissue enrichment analysis

We determined the enriched tissues for pleiotropic variants using stratified LDSC (S-LDSC)^[27]^ and pleiotropic genes using MAGMA gene property tests^[28]^, respectively.

S-LDSC is based on a hypothesis that SNPs with high LD to a category have a higher χ^2^ than SNPs with low LD to this category, thereby indicating the category's heritability enrichment. Specifically,

1 $ E\left[{\chi }_{j}^{2}\right]=N{\sum }_{c}{\tau }_{c}l\left(j,C\right)+Na+1 . $

$ E\left[{\chi }_{j}^{2}\right] $ is the expected statistics of SNP $ j $, $ N $ is the sample number, $ C $ represents categories, $ l\left(j,C\right) $ is the LD score of SNP $ j $ in category $ C $, and $ a $ is the confounder. A total of 220 tissue- and cell-type-specific annotations were pre-defined based on four histone modification markers: H3K27ac, H3K9ac, H3K4me3, and H3K4me1^[27]^. The enriched tissues and cell types were identified if the coefficient *P* < 2.27 × 10^−4^ (0.05/220).

MAGMA gene property test is based on a linear regression model,

2 $ Z~{\beta }_{0}+{{ E}}_{t}{\beta }_{t}+A{\beta }_{{\rm A}}+B{\beta }_{B}+\varepsilon . $

$ Z $ is transformed from the MAGMA *P* of the pleiotropic genes, $ {{ E}}_{t} $ is the gene expression of a specific tested tissue type, $ A $ is the average gene expression of all tissues, and $ B $ is the confounder. The gene expression matrix of 54 tissues from Genotype-Tissue Expression version 8^[8]^ was used as a reference for MAGMA gene property tests, with enriched tissues declared at an associated *P* < 9.26 × 10^−4^ (0.05/54).

### Protein-protein interaction (PPI) analysis

The reference PPI panel was constructed with the STRING database^[29]^, with self-links and low-confidence (combined score < 0.7) proteins excluded, resulting in 16758 nodes and 411585 edges. Then, we included pleiotropic genes (excluding non-protein-coding genes and genes without Entrez identifiers) in the reference panel to generate the GSA PPI network (GSA-PPIN).

Since proteins typically function as modules^[30]^, we used the Louvain algorithm^[31]^ to detect functional modules within the GSA-PPIN, assessing their significance using the qs-test^[32]^ with 10000 randomly generated networks. Gene Ontology biological process and Kyoto Encyclopedia of Genes and Genomes (KEGG) enrichment analyses were performed on each module to identify associated biological pathways using the clusterProfiler package^[33]^. Visualization of the PPI network was conducted with Cytoscape^[34]^.

### Mendelian randomization and mediation analysis

Bidirectional MR analysis was performed to detect the causal associations between 28 pairs of skin and GIT diseases. Instrumental variables (IVs) were selected as variants with *P* < 5 × 10^−8^ in exposure GWAS and LD *r*^2^ < 0.01 in a 10 Mb window, based on the 1000 Genomes Project Phase 3 Europeans^[19]^. IVs associated with skin or GIT diseases other than exposure or outcome, failing the Steiger test^[35]^, or identified MR-PRESSO^[36]^ outliers were excluded. Inverse-variance weighted (IVW) estimates^[37]^ were reported as the primary MR results, while MR-Egger^[38]^, weighted median^[39]^, weighted mode^[40]^, RAPS^[41]^, and BWMR^[42]^ were used as sensitivity analyses. Statistical significance was declared at *P* < 8.93 × 10^−4^ (0.05/56).

Given the crucial role of gut microbiota in GIT diseases, we further investigated its role in the GSA *via* two-stage MR and mediation analyses for significant causal associations from GIT diseases to skin diseases. In the first stage, the causal effects of the microbiota on GIT diseases and skin diseases were estimated, and in the second stage, the effects of GIT diseases on skin diseases were evaluated. Specifically, IVs for microbiota were selected as variants with *P* < 1 × 10^−5^ in microbiome GWASs (430 microbiome features from 8956 German individuals)^[43]^ and LD *r*^2^ < 0.001 in a 10-Mb window, based on the 1000 Genomes Project Phase 3 Europeans^[19,44]^. Mediation effects were calculated as the product of the microbiota's effects on GIT diseases and the effect of GIT diseases on skin diseases, with standard errors estimated using the delta method^[45]^.

## Results

### Genetic correlation between skin and GIT diseases

Among the 28 pairs of skin and GIT diseases, 22 pairs were nominally significant in at least one global genetic correlation method, and 19 pairs were significant in both. After multiple testing corrections, 15 pairs remained significant in at least one method, and 8 in both (***Table 1***). All 22 nominally significant trait pairs showed positive genetic correlations, ranging from 0.074 to 0.542, suggesting that skin diseases are genetically associated with severe gastrointestinal disorders.

**Table 1:** Genetic correlation between skin and GIT diseases

Trait pairs	LDSC			ρ-HESS	
	*r*_*g*_ (SE)	*P*_*LDSC*_		*r*_*g*_ (SE)	*P*_*ρ-HESS*_
AD–PUD	0.181 (0.071)	0.010		0.074 (0.034)	0.031
AD–GORD	0.152 (0.044)	**6.04 × 10** ^**−4**^		0.100 (0.021)	**2.25 × 10** ^**−6**^
AD–IBD	0.157 (0.070)	0.024		0.127 (0.020)	**3.23 × 10** ^**−10**^
AD–IBS	0.100 (0.045)	0.026		0.119 (0.025)	**2.13 × 10** ^**−6**^
HS–PUD	0.379 (0.139)	0.006		0.149 (0.076)	0.049
HS–GORD	0.450 (0.107)	**2.75 × 10** ^**−5**^		0.264 (0.043)	**6.30 × 10** ^**−10**^
HS–IBD	0.239 (0.080)	0.003		0.115 (0.038)	0.003
HS–IBS	0.193 (0.080)	0.016		0.191 (0.057)	**7.23 × 10** ^**−4**^
Acne–PUD	−0.094 (0.062)	0.130		−0.086 (0.103)	0.404
Acne–GORD	0.068 (0.042)	0.105		0.059 (0.062)	0.346
Acne–IBD	0.136 (0.049)	0.006		0.195 (0.069)	0.005
Acne–IBS	0.148 (0.052)	0.005		0.273 (0.103)	0.008
SLE–PUD	0.068 (0.093)	0.466		−0.034 (0.046)	0.457
SLE–GORD	0.120 (0.050)	0.016		−0.025 (0.036)	0.491
SLE–IBD	0.178 (0.050)	**3.75 × 10** ^**−4**^		0.049 (0.029)	0.091
SLE–IBS	0.136 (0.075)	0.069		0.075 (0.043)	0.085
Psoriasis–PUD	0.250 (0.072)	**5.21 × 10** ^**−4**^		0.110 (0.031)	**4.05 × 10** ^**−4**^
Psoriasis–GORD	0.186 (0.045)	**2.92 × 10** ^**−5**^		0.089 (0.018)	**1.35 × 10** ^**−6**^
Psoriasis–IBD	0.265 (0.049)	**8.24 × 10** ^**−8**^		0.170 (0.018)	**1.55 × 10** ^**−20**^
Psoriasis–IBS	0.130 (0.044)	0.003		0.096 (0.021)	**6.95 × 10** ^**−6**^
Rosacea–PUD	0.214 (0.141)	0.130		0.219 (0.140)	0.118
Rosacea–GORD	0.458 (0.123)	**1.86 × 10** ^**−4**^		0.363 (0.105)	**5.46 × 10** ^**−4**^
Rosacea–IBD	0.457 (0.108)	**2.34 × 10** ^**−5**^		0.542 (0.244)	0.026
Rosacea–IBS	0.048 (0.099)	0.629		0.573 (0.416)	0.168
Urticaria–PUD	0.326 (0.090)	**2.96 × 10** ^**−4**^		0.165 (0.067)	0.014
Urticaria–GORD	0.324 (0.064)	**4.89 × 10** ^**−7**^		0.240 (0.046)	**2.18 × 10** ^**−7**^
Urticaria–IBD	0.098 (0.059)	0.096		0.089 (0.037)	0.016
Urticaria-IBS	0.322 (0.064)	**5.53 × 10** ^**−7**^		0.288 (0.058)	**7.81 × 10** ^**−7**^
The genetic correlation of 28 trait pairs (seven skin diseases × four GIT diseases) was estimated from the global level with LDSC and on the local scale with ρ-HESS. ρ represents local genetic correlation. The local genetic correlations in ρ-HESS were summed to derive the global genetic correlation. Significant results (*P* < 0.0018; 0.05/28) were marked in bold. Abbreviations: AD, atopic dermatitis; GORD, gastro-oesophageal reflux disease; HESS, heritability estimation from summary statistics; HS, hidradenitis suppurativa; IBD, inflammatory bowel disease; IBS, irritable bowel syndrome; LDSC, linkage disequilibrium score regression; PUD, peptic ulcer disease; SLE, systemic lupus erythematosus.					

At the local scale, 636 significant regions (566 unique) were identified across six trait pairs, including AD–IBD, SLE–PUD, SLE–GORD, SLE–IBD, SLE–IBS, and psoriasis–IBD (***Supplementary Fig. 1***; ***Supplementary Table 2***). Among regions, 57 showed local genetic correlations for multiple trait pairs, with the major histocompatibility complex (MHC) region being particularly prominent. Specifically, chr6:31571218–32682664 and chr6:32682664–33236497 were identified in five trait pairs, while chr6:29737971–30798168 and chr6:30798168–31571218 were implicated in four trait pairs. Beyond the MHC region, three additional regions were identified across three trait pairs, including chr1:99800604–100826405, chr1:118839067–144977494, and chr8:136035605–137524151.

### Pleiotropic loci between skin and GIT diseases

We found a total of 27019 pleiotropic variants (15803 unique) between skin and GIT diseases (***Supplementary Figs. 2*** and ***3***). These were merged into 298 pleiotropic loci, including 282 unique lead variants, and annotated to 217 nearest genes (***Supplementary Table 3***). Among the lead variants, 135 (45.30%) were intergenic, and 122 (40.94%) intronic. Only 19 (6.38%) exonic, including four non-coding RNA exonic variants and 15 (12 unique) messenger RNA exonic variants (***Supplementary Table 4***). Notably, several functionally significant variants were identified: rs12720356 in *TYK2*, rs1336900 in *HORMAD1*, rs1990760 in *IFIH1*, rs33980500 in *TRAF3IP2*, and rs56094005 in *DOK2* are missense variants; rs601338 in *FUT2* is a stop-gain variant; and rs8176719 in *ABO* is a frameshift variant.

**Figure 2:**
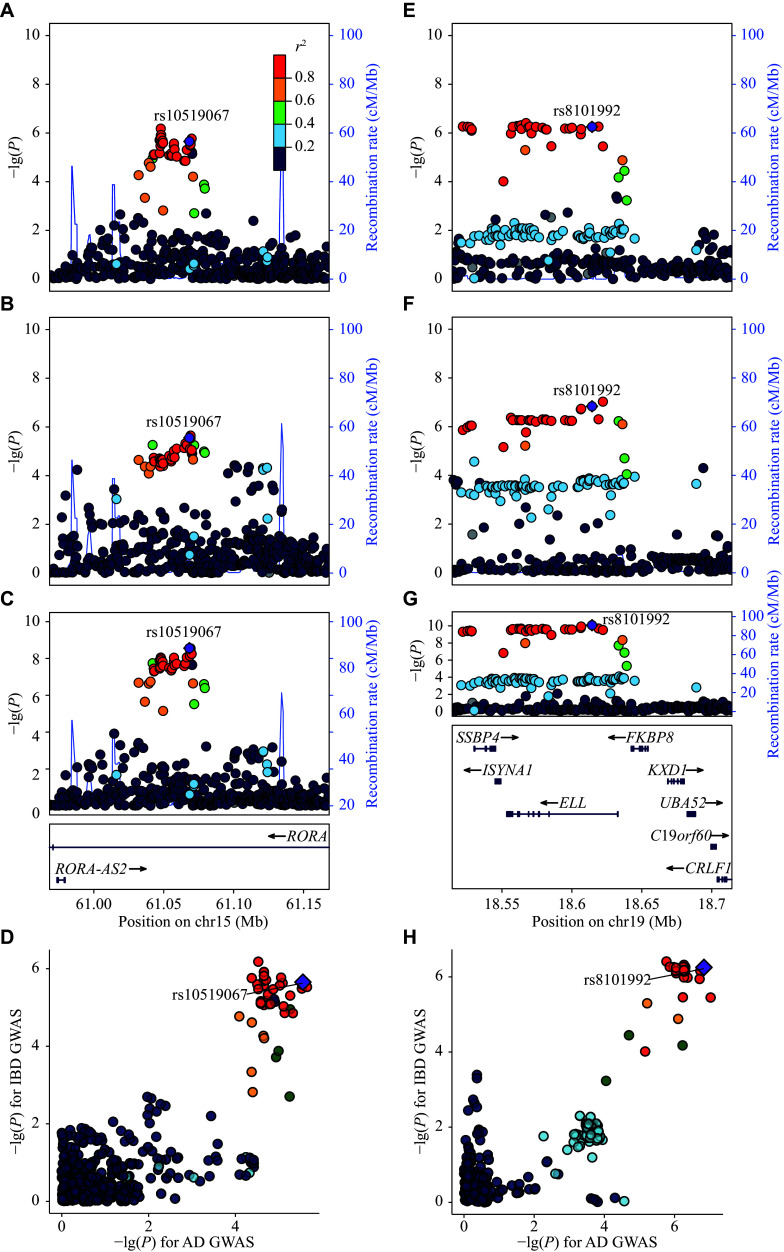
Novel loci identified for AD–IBD based on the pleiotropic analysis. A–D: rs10519067 (*RORA*, 15q22.2). E–H: rs8101992 (*ELL*, 19p13.11). From top to bottom, regional plots for IBD GWAS, AD GWAS, and PLACO analysis, as well as a comparison of single-trait GWAS statistics, are shown. The lead variants in PLACO analysis are colored in purple. The other variants are colored according to their LD to the lead variant. Abbreviations: AD, atopic dermatitis; GWAS, genome-wide association study; IBD, inflammatory bowel disease; LD, linkage disequilibrium; PLACO, pleiotropic analysis under composite null hypothesis.

Notably, 61 loci showed pleiotropic effects across multiple trait pairs, including *PUS10* at 2p16.1, *HLA-DRB1* at 6p21.32, *ZNF365* at 10q21.2, and *SLC7A10* at 19q13.11, each identified in four trait pairs (***Supplementary Table 3***). Additionally, five novel loci were identified: *RORA* at 15q22.2 (rs10519067, *P* = 2.76 × 10^−9^) and *ELL* at 19p13.11 (rs8101992, *P* = 8.34 × 10^−11^) in AD–IBD (***Fig. 2***), *LOC285819* at 6p22.2 (rs11432623, *P* = 6.08 × 10^−10^) in AD–IBS (***Supplementary Fig. 4R***), *TAMM41* at 3p25.2 (rs62246110, *P* = 4.69 × 10^−8^) in HS–IBD (***Supplementary Fig. 4V***), and *LOC101928370* at 1p21.2 (rs74665711, *P* = 1.31 × 10^−8^) in rosacea–PUD (***Supplementary Fig. 4BR***). Notably, *RORA* at 15q22.2 was verified in the latest cross-population GWAS meta-analysis of IBD^[46]^.

### Colocalization of pleiotropic loci in skin and GIT diseases

Among the 298 pleiotropic loci, 75 (25.17%) showed colocalization for pairs of skin and GIT diseases, of which 56 (74.67%) had lead variants with effects in the same direction, suggesting that these variants may simultaneously increase or decrease the risk of both skin and GIT diseases (***Fig. 2***; ***Supplementary Fig. 4***; ***Supplementary Table 3***). Notably, rs938650 (8q24.21) was colocalized in both AD–IBD (PP4 = 0.921) and psoriasis–IBD (PP4 = 0.948); rs1469149 (5q31.1) was colocalized in both psoriasis-IBD (PP4 = 0.991) and rosacea–IBD (PP4 = 0.974); and rs11066188 (12q24.13) was colocalized in both AD–IBD (PP4 = 0.998) and SLE–IBD (PP4 = 0.999). In contrast, the missense variant rs1990760 in *IFIH1* was only colocalized in SLE–IBD (PP4 = 0.941) but not in psoriasis–IBD (PP4 = 0.211), and the frameshift variant rs8176719 in *ABO* was colocalized only in psoriasis–PUD (PP4 = 0.930) but not in HS–PUD (PP4 = 0.628).

Additionally, all five novel loci were colocalized, with lead variants being the most likely causal variants. Specifically, rs10519067 was colocalized in AD–IBD (PP4 = 0.946), rs8101992 was colocalized in AD–IBD (PP4 = 0.967), rs11432623 was colocalized in AD–IBS (PP4 = 0.865), rs62246110 was colocalized in HS–IBD (PP4 = 0.795), and rs74665711 was colocalized in rosacea–PUD (PP4 = 0.817).

### Druggable pleiotropic genes for skin and GIT diseases

Based on PLACO results, 1184 pleiotropic genes (394 unique) were identified using MAGMA, with 214 genes associated with multiple trait pairs (***Supplementary Table 5***). Among them, *MSH5–SAPCD1*, *MSH5*, and *ATP6V1G2* were implicated in 16 trait pairs, followed by *LSM2* and *HLA-DOB* in 15 trait pairs.

According to DrugBank, 66 pleiotropic genes were druggable (***Supplementary Table 6***), of which 18 are targeted by drugs for skin or GIT diseases (***Table 2***). For example, lebrikizumab (targeting *IL13*) is indicated for AD; efalizumab (targeting *ITGAX*) is applicable to psoriasis; and ozanimod (targeting *S1PR5*) is suitable for ulcerative colitis. Notably, ustekinumab and risankizumab (both targeting *IL12B*) as well as adalimumab and infliximab (both targeting *TNF*), have already been approved for treating both skin and GIT diseases.

**Table 2:** Pleiotropic genes druggable for skin diseases or GIT diseases

Pleiotropic genes	Drugs	Indication	Action	Trait pairs
*CYP21A2*	Ketoconazole	Seborrheic dermatitis and fungal skin infections	Inhibitor	AD–IBD, HS–IBD, SLE–IBD, SLE–IBS, Urticaria–IBD
*ERBB2*	Trastuzumab	Gastroesophageal and gastric cancers	Binder antibody	SLE–IBD
*ERBB3*	Tucatinib	Colorectal cancer	Inhibitor	AD–GORD
*FCGR2A*	Etanercept	Plaque psoriasis	Ligand	SLE–IBD
*FCGR2B*	Etanercept	Plaque psoriasis	Ligand	SLE–IBD
*IL12B*	**Ustekinumab**	Plaque psoriasis, Crohn's disease, and ulcerative colitis	Inhibitor	Psoriasis–IBD
*IL13*	Lebrikizumab	Atopic dermatitis	Antibody	AD–PUD, Psoriasis–PUD, Psoriasis–IBD
*IL2*	Cefazolin	Bacterial infections of the skin	Inhibitor	AD–IBD, AD–IBS, Psoriasis–IBD
*IL2RA*	Aldesleukin	Metastatic melanoma	Agonist modulator	AD–PUD, AD–IBD
*IL4*	Dupilumab	Atopic dermatitis	Inhibitor antibody	AD–GORD, AD–IBD, Psoriasis–IBD
*ITGAX*	Efalizumab	Chronic plaque psoriasis	Antibody	SLE–IBD
*KAT5*	Coenzyme A	Acne (in trials)	Unknown^*^	AD–IBD, AD–IBS
*LTA*	Etanercept	Plaque psoriasis	Ligand	AD–PUD, AD–GORD, SLE–PUD, SLE–GORD, SLE–IBD, SLE–IBS, Psoriasis–PUD, Psoriasis–GORD, Psoriasis–IBD, Urticaria–GORD
*NEU1*	Celecoxib	Familial adenomatous polyposis	Inhibitor	AD–IBD, SLE–IBD, Urticaria–GORD, Urticaria–IBS
*PDE4A*	Roflumilast	Psoriasis vulgaris and seborrheic dermatitis	Inhibitor	SLE–IBD, Psoriasis–IBD
*S1PR5*	Ozanimod	Ulcerative colitis	Agonist	SLE–IBD, Psoriasis–IBD
*TNF*	**Adalimumab**	Crohn's disease, ulcerative colitis, psoriasis, and hidradenitis suppurativa	Inhibitor antibody	Psoriasis–IBS
*TUBB*	Tirbanibulin	Actinic keratosis	Inhibitor	Acne–GORD, Urticaria–GORD, Urticaria–IBD, Urticaria–IBS
For each gene, only one drug is present (All drugs are listed in ***Supplementary Table 6***). Drugs simultaneously applicable to skin diseases and GIT diseases are marked in bold.*Unknown means still in trials.Abbreviations: AD, atopic dermatitis; GORD, gastro-oesophageal reflux disease; HS, hidradenitis suppurativa; IBD, inflammatory bowel disease; IBS, irritable bowel syndrome; PUD, peptic ulcer disease; SLE, systemic lupus erythematosus.				

### Pleiotropy enriched in GSA-associated tissues and the immune system

**Figure 3:**
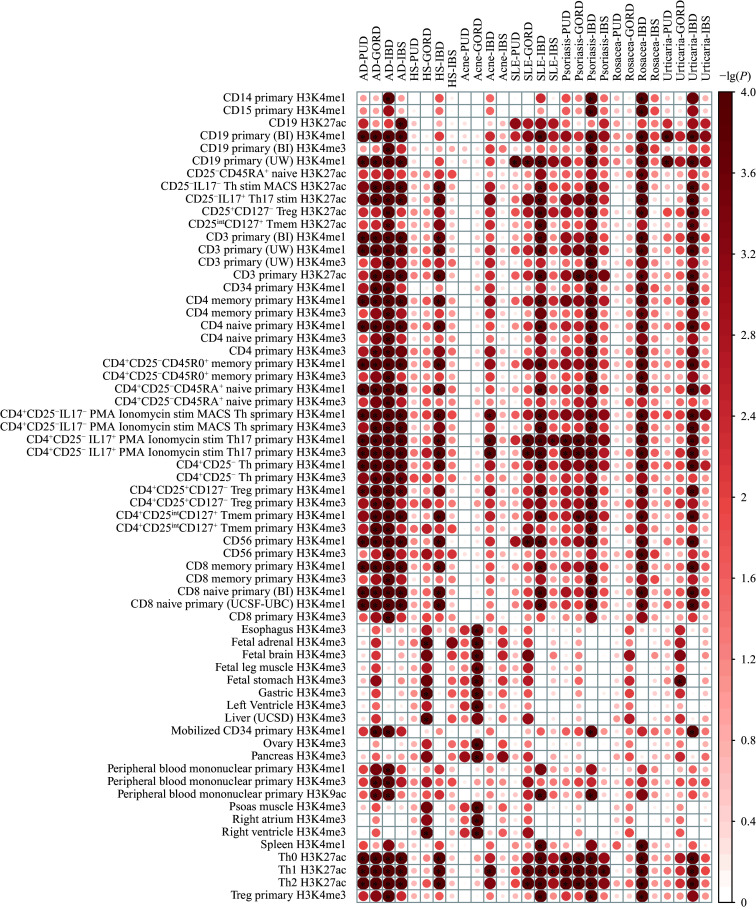


Based on S-LDSC, 330 significant heritability enrichments were identified across 64 of 220 tissue- and cell-type-specific annotations (***Fig. 3A***; ***Supplementary Table 7***). Among these 330 significant enrichments, 178 (53.94%) were marked by H3K4me1 histone modification. In addition to GSA-associated tissues, such as the esophagus, gastric tissue, and fetal stomach, heritability was also enriched in immune cells, with Th cells being the top-enriched cell type for 12 of 28 trait pairs. MAGMA gene property analyses further highlighted enrichment in GSA-associated tissues, including the transverse colon and terminal ileum of the small intestine, as well as immune-related tissues such as EBV-transformed lymphocytes, spleen, and whole blood (***Fig. 3B***).

### Pleiotropic modules and pathways between skin and GIT diseases

After filtering out non-protein-coding genes or genes lacking Entrez identifiers, 377 pleiotropic genes remained. Among these, the GSA-PPIN comprised 215 nodes (genes) and 494 edges (interactions) (***Supplementary Fig. 5***; ***Supplementary Table 8***). Using the Louvain module algorithm, four significant modules were identified, each corresponding to distinct biological pathways (***Supplementary Fig. 5***; ***Supplementary Tables 8*** and ***9***). Module One is the GPCR-cAMP signaling pathway. Module Two represents chromatin remodeling, a major type of epigenetic modification. The largest module, Module Three, is the JAK-STAT pathway and includes 11 of 18 (61%) aforementioned druggable genes targeting skin or GIT diseases (***Table 2***). The last module represents HLA-mediated immunity. GO and KEGG enrichment analyses further supported these findings (***Supplementary Fig. 6***).

### Causal relationships between skin and GIT diseases

**Figure 4:**
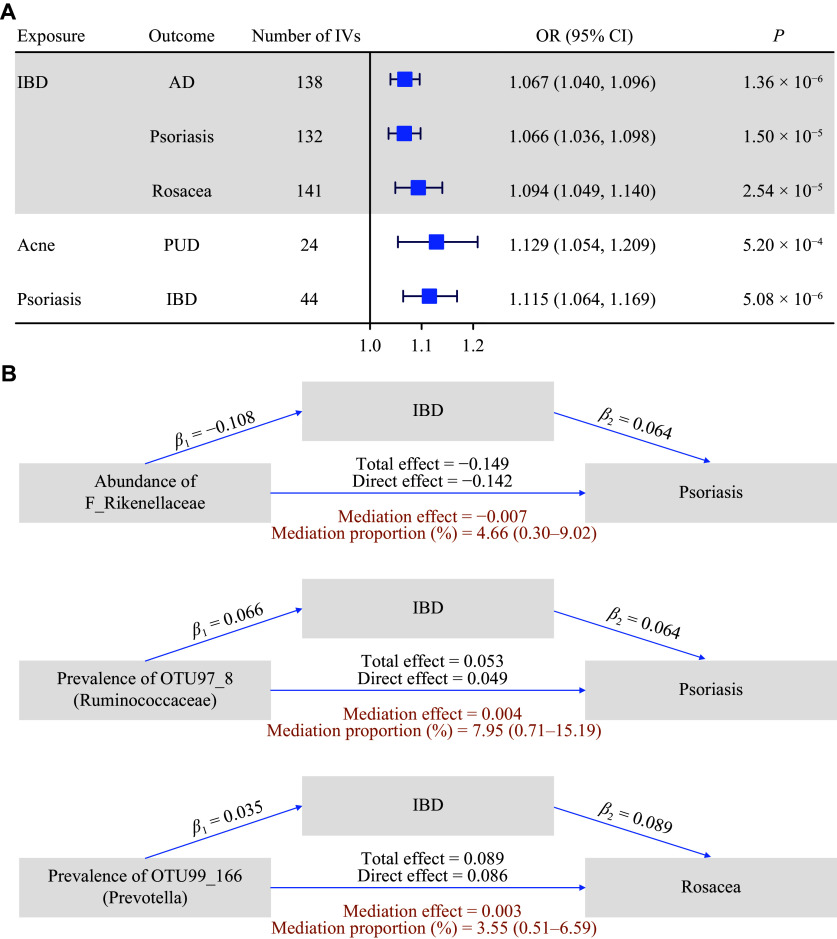
Causal inference of skin and GIT diseases. A: Significant causal associations identified with IVW in bidirectional-MR (all bidirectional-MR results are listed in ***Supplementary Tables 10*** and ***11***. OR (95% CI) is the odds ratio with a corresponding 95% confidence interval. The significance was declared at *P* < 8.93 × 10^−4^ (0.05/56). B: Potential microbiota-GIT-skin pathways identified with IVW in two-stage MR (***Supplementary Tables 12*** and ***13***). Stage one estimated the effects of microbiota on GIT diseases (*β*_1_), and stage two estimated the effects of microbiota on skin diseases (total effect). The mediation effect was calculated as the product of *β*_1_ and *β*_2_ (the effect of GIT diseases on skin diseases). The significance of each step was declared at *P* < 0.05. Abbreviations: AD, atopic dermatitis; CI, confidence interval; GIT, gastrointestinal tract; IBD, inflammatory bowel disease; IVs, instrumental variables; IVW, inverse-variance weighted; MR, Mendelian randomization; OR, odds ratio.

We identified five significant causal associations using the IVW method: three from GIT diseases to skin diseases (*i.e.*, IBD to AD, IBD to psoriasis, and IBD to rosacea) and two from skin diseases to GIT diseases (*i.e.*, acne to PUD and psoriasis to IBD) (***Fig. 4A***). Notably, a bidirectional causal association between IBD and psoriasis was found. All these significant causal associations were positive. Results from sensitivity analyses were largely consistent with those of the IVW method (***Supplementary Tables 10*** and ***11***).

Moreover, in mediation analyses of gut microbiota, we identified three potential pathways: *Rikenellaceae*-IBD-psoriasis, *Ruminococcaceae*-IBD-psoriasis, and *Prevotella*-IBD-rosacea (***Fig. 4B***; ***Supplementary Tables 12*** and ***13***). *Rikenellaceae* exhibited potential protective effects for both skin and GIT diseases. However, the mediation accounted for only 4.66%, 7.95%, and 3.55%, respectively.

## Discussion

In this genome-wide pleiotropic analysis, we found extensive genetic correlations and overlaps between skin and GIT diseases. Specifically, pleiotropy was widely distributed across the variant, gene, and pathway levels. Not only were all significant genetic correlations and causal relationships positive, but most lead variants at colocalized loci showed concordant associations in the original GWASs. These findings further suggest that skin and GIT diseases may mutually influence each other *via* the GSA.

Global genetic correlations were identified across multiple trait pairs, while local genetic correlations were predominantly identified in SLE and IBD (***Supplementary Table 2***). This may be attributed to their high heritability (***Supplementary Table 1***). Clinically, individuals with SLE were more likely to develop GIT diseases than those with other skin diseases, and IBD patients were more prone to comorbid skin diseases than those with other GIT diseases^[47]^.

Pleiotropic loci between skin and GIT diseases were widely distributed, with several lead variants being protein-altering (***Supplementary Discussion***). Notably, 61 loci were identified in multiple trait pairs, such as 2p16.1 (*PUS10*), 6p21.32 (*HLA-DRB1*), 10q21.2 (*ZNF365*), and 19q13.11 (*SLC7A10*). Additionally, five novel loci were identified, with 15q22.2 (*RORA*) validated by the latest cross-population meta-analysis of IBD GWAS^[46]^ (***Supplementary Discussion***). The pleiotropic loci were highly enriched in GSA-associated tissues and the immune system.

Shared genetic factors also manifest as shared biological pathways and extensive causal associations (***Supplementary Discussion***). A total of four modules were identified using PPI analysis. Notably, Module Three (the JAK-STAT pathway) showed strong potential for drug repurposing, as adalimumab and infliximab (targeting *TNF*) and ustekinumab and risankizumab (targeting *IL-12B*) have already been approved to treat both skin and GIT diseases. Although each module corresponds to distinct pathways, including signal transduction, epigenetic reprogramming, non-HLA-mediated immunity, and HLA-mediated immunity, they collectively contribute to GSA comorbidity.

Our study has several limitations. First, our analysis was based on GWAS summary statistics, which did not allow for standardized quality control as individual data. Second, the statistical power for HS (1070 cases and 394105 controls) and rosacea (2455 cases and 394105 controls) may be limited due to the relatively small number of cases. Third, we included only European populations, limiting the generalizability to other populations. Replication in diverse populations is needed.

In conclusion, the current study demonstrates that extensive genetic correlations, genetic overlaps, and causal relationships exist between skin and GIT diseases. These findings support a shared genetic basis of GSA and provide novel insights into strategies for their simultaneous management.

## SUPPLEMENTARY DATA

Supplementary data to this article can be found online.
