# Supplementary material for: Cross-phenotype genome-wide association study supports shared genetic etiology between skin and gastrointestinal tract diseases
Source: J Biomed Res. 2026 Mar 19;40(2):172–84. doi: 10.7555/JBR.39.20250166 (PMC13044403; doi:10.7555/JBR.39.20250166)
Supplement: Supplementary file 1 — Supplementary data to this article can be found online. [file jbr-40-2-172-S1.pdf]

Available online at www.jbr-pub.org.cn

Open Access at PubMed Central

JBR

Journal of Biomedical Research, 2026 40(2): 1–34

Supplementary Data

Cross-phenotype genome-wide association study supports shared genetic etiology between skin and gastrointestinal tract diseases

Bo Peng[1,2], Minghui Jiang[3,4], Si Li[1,2], Xingyu Chen[1,2], Shanshan Cheng[1,2,✉], Xingjie Hao[1,2,✉]

[1]*Department of Epidemiology and Biostatistics, School of Public Health, Tongji Medical College, Huazhong University of Science and Technology, Wuhan, Hubei 430030, China;*
[2]*Ministry of Education Key Laboratory of Environment and Health, School of Public Health, Tongji Medical College, Huazhong University of Science and Technology, Wuhan, Hubei 430030, China;*
[3]*Department of Neurology, Beijing Tiantan Hospital, Capital Medical University, Beijing 100070, China;*
[4]*China National Clinical Research Center for Neurological Diseases, Beijing 100070, China.*

Supplementary Discussion

Extensive genetic overlaps were identified in pleiotropic analyses. Several lead variants at pleiotropic loci are located in exons. *IFIH1* encodes an RNA helicase crucial for recognizing exogenous RNA[1]. The missense variant rs1990760 in *IFIH1* may lead to misrecognition of endogenous RNA as exogenous[2–3]. Although this variant was identified in two trait pairs (P_{PLACO} = 1.91 × 10^{-9} for SLE–IBD and P_{PLACO} = 2.24 × 10^{-10} for psoriasis–IBD), it colocalized only in SLE–IBD (PP4 = 0.941; ***Supplementary Fig. 4AH***) rather than psoriasis–IBD (PP4 = 0.211), suggesting potentially distinct mechanisms of *IFIH1* in different gut–skin axis (GSA)-associated diseases. Additionally, the stop-gain variant rs601338 in *FUT2* and the frameshift variant rs8176719 in *ABO* may exhibit functional interaction. *FUT2*, known as a "secretory gene", plays an important role in GIT diseases by regulating the secretion of the H antigen encoded by *ABO*[4–6]. The H antigen is a secretory carbohydrate antigen and can be utilized by certain bacteria, which affects the occurrence and progression of GIT diseases[7–9]. Individuals with type O blood, primarily determined by rs8176719 in *ABO*, or with rs601338 in *FUT2* will have less secretion of H antigen[7–9].

PLACO can detect novel shared loci that are not identified by the original GWAS[10]. Here, five novel loci were identified between skin and GIT diseases, including rs10519067 at 15q22.2 and rs8101992 at 19p13.11 in AD–IBD, rs11432623 at 6p22.2 in AD–IBS, rs62246110 at 3p25.2 in HS–IBD, and rs74665711 at 1p21.2 in rosacea–PUD. All novel loci were colocalized, with lead variants being the most potentially causal variants. Specifically, rs10519067 is an intronic variant in *RORA*, a transcription factor crucial for Th cell differentiation and stability[11], which is highly expressed in active UC patients[12]. Notably, this locus has been confirmed in the latest cross-population meta-analysis of IBD GWAS[13]. The intronic variant rs8101992 is a significant eQTL of *ELL* in multiple tissues, including skin and esophagus (***Supplementary Fig. 7***, available online). *ELL*, which encodes an RNA polymerase Ⅱ elongation factor with E3 ubiquitin ligase activity, is essential for skin proliferation[14]. rs11432623, an intronic variant in ncRNA *LOC285819*, serves as a significant eQTL for *BTN3A2* in almost all tissue types (***Supplementary Fig. 7***). *BTN3A2* encodes a member of the immunoglobulin superfamily[15–16]. Its paralog *BTN3A3*, which

✉Corresponding authors: Xingjie Hao and Shanshan Cheng, Department of Epidemiology and Biostatistics, School of Public Health, Tongji Medical College, Huazhong University of Science and Technology, 13 Hangkong Road, Wuhan, Hubei 430030, China. E-mails: xingjie@hust.edu.cn (Hao) and sscheng@hust.edu.cn (Cheng).

Received: 19 February 2025; Revised: 19 May 2025; Accepted: 22 May 2025; Available online: 28 May 2025; Published date: 19 March 2026

CLC number: R394, Document code: A

The authors reported no conflict of interests.

This is an open access article under the Creative Commons Attribution (CC BY 4.0) license, which permits others to distribute, remix, adapt and build upon this work, for commercial use, provided the original work is properly cited.

© 2026 by Journal of Biomedical Research.

https://doi.org/10.7555/JBR.39.20250166

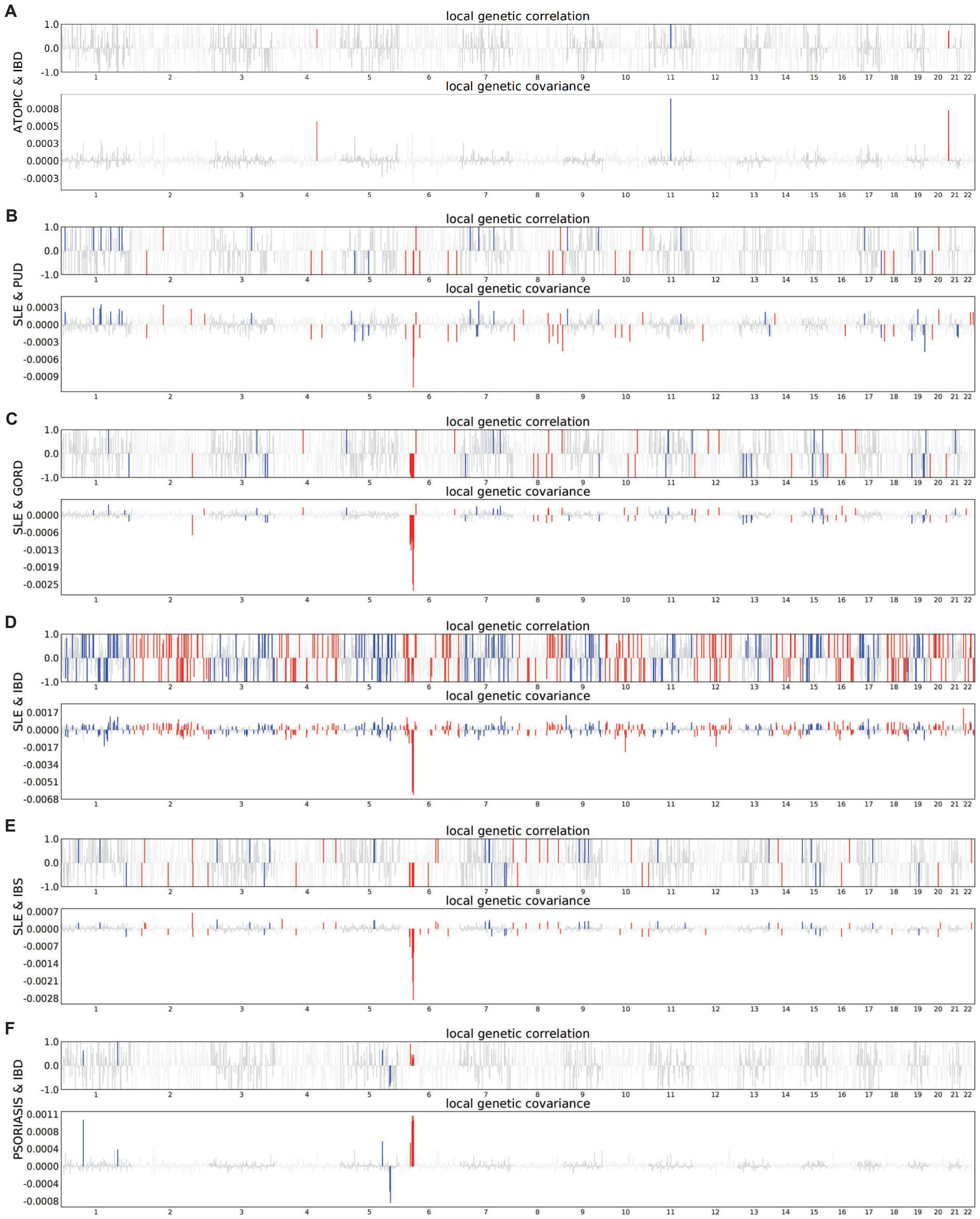

Supplementary Fig. 1 **Local genetic correlation between skin and gastrointestinal tract diseases.** A: SLE–PUD. B: SLE–GORD. C: SLE–GORD. D: SLE–IBD. E: SLE–IBS. F: Psoriasis–IBD. The red and blue lines represent significant positive and negative correlations in a linkage disequilibrium block, respectively. The significance was declared at $P < 2.94 \times 10^{-5}$ (0.05/1 703). Abbreviations: GORD, gastro-oesophageal reflux disease; IBD, inflammatory bowel disease; IBS, irritable bowel syndrome; PUD, peptic ulcer disease; SLE, systemic lupus erythematosus.

plays an important role in γδT cell activation[17–18], has been associated with psoriasis susceptibility[19]. rs62246110 is a variant located upstream of *TAMM41*. *TAMM41* encodes an inner mitochondrial membrane protein that is underexpressed in colorectal cancer[20]. rs74665711 is an intergenic variant located between *LOC101928370* and *LOC102606465*, with the nearest protein-coding gene being *S1PR1*. *S1PR1* modulates

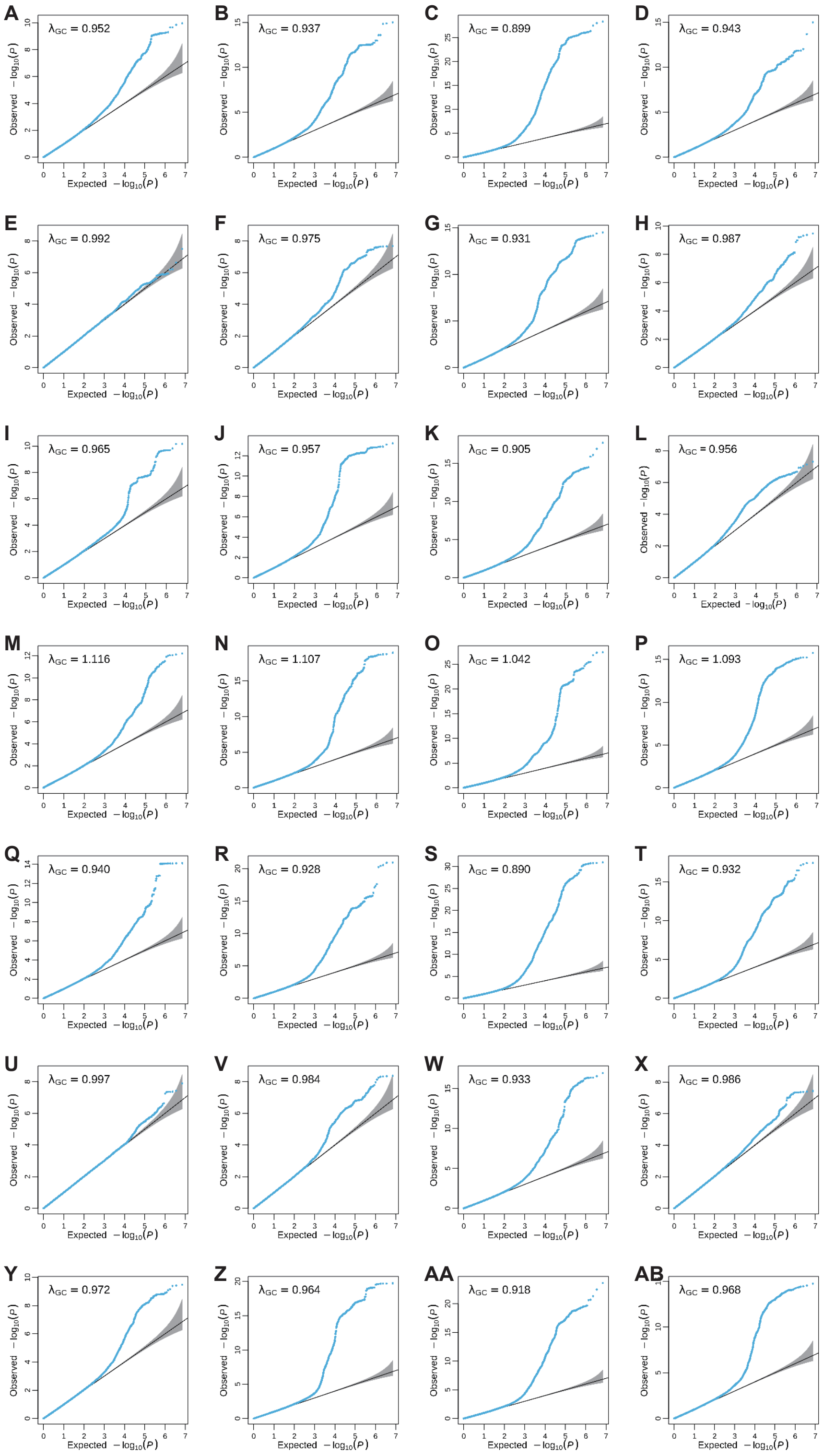

Supplementary Fig. 2 **QQ plots for PLACO analyses.** A: AD–PUD. B: AD–GORD. C: AD–IBD. D: AD–IBS. E: HS–PUD. F: HS–GORD. G: HS–IBD. H: HS–IBS. I: Acne–PUD. J: Acne–GORD. K: Acne–IBD. L: Acne–IBS. M: SLE–PUD. N: SLE–GORD. O: SLE–IBD. P: SLE–IBS. Q: Psoriasis–PUD. R: Psoriasis–GORD. S: Psoriasis–IBD. T: Psoriasis–IBS. U: Rosacea–PUD. V: Rosacea–GORD. W: Rosacea–IBD. X: Rosacea–IBS. Y: Urticaria–PUD. Z: Urticaria–GORD. AA: Urticaria–IBD. AB: Urticaria–IBS. The x-axis shows the expected $-\lg(P_{\mathrm{PLACO}})$, and the y-axis shows the observed $-\lg(P_{\mathrm{PLACO}})$.

immune cell differentiation and migration by activating intracellular signaling through S1P binding[21]. *S1PR1* inhibitors, like fingolimod, exhibit potential therapeutic effects in IBD by inhibiting T cell migration and inflammation[22]. Besides, *S1PR5*, a subfamily member, is involved in the Module One GPCR-cAMP signaling pathway.

To further elucidate the potential mechanisms of

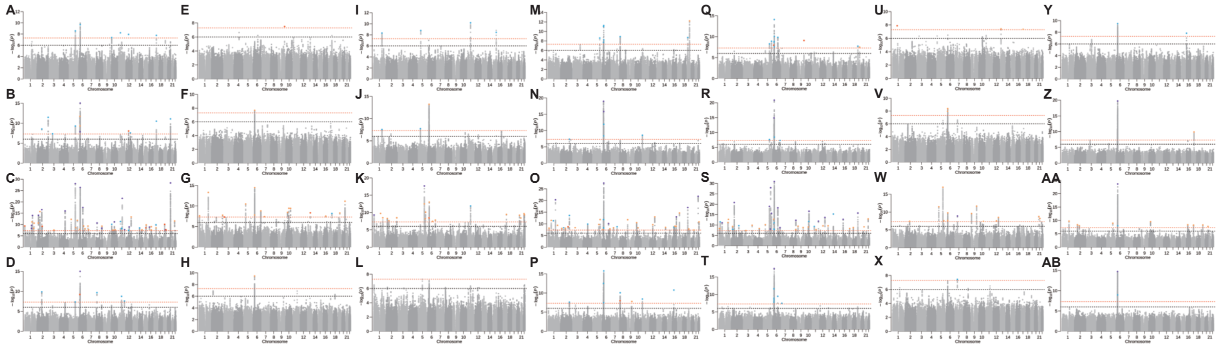

Supplementary Fig. 3 **Manhattan plots for PLACO analyses.** A: AD–PUD. B: AD–GORD. C: AD–IBD. D: AD–IBS. E: HS–PUD. F: HS–GORD. G: HS–IBD. H: HS–IBS. I: Acne–PUD. J: Acne–GORD. K: Acne–IBD. L: Acne–IBS. M: SLE–PUD. N: SLE–GORD. O: SLE–IBD. P: SLE–IBS. Q: Psoriasis–PUD. R: Psoriasis–GORD. S: Psoriasis–IBD. T: Psoriasis–IBS. U: Rosacea–PUD. V: Rosacea–GORD. W: Rosacea–IBD. X: Rosacea–IBS. Y: Urticaria–PUD. Z: Urticaria–GORD. AA: Urticaria–IBD. AB: Urticaria–IBS. The x-axis shows the position of chromosomes, and the y-axis shows $-\lg(P_{PLACO})$. The red dashed lines represent the significant level of 5×10^{-8}, and the dark dashed lines represent the suggestive level of 1×10^{-6}. The purple dots indicate the significance in both GIT GWAS and skin GWAS, the orange dots indicate the significance only in GIT GWAS, the blue dots indicate the significance only in skin GWAS, and the red dots suggest that the variants were significant in neither GWAS trait.

GSA, we performed protein–protein interaction (PPI) analysis on pleiotropic genes, identifying four significant modules in the GSA-associated PPI network (GSA-PPIN). Module One corresponds to the GPCR-cAMP pathway, which is involved in identifying xenobiotics[23–24], such as bacterial invaders[25]. Both skin and GIT are barrier tissues rich in microbiota, crucial for protecting against exogenous infections[26]. Module Two highlights chromatin remodeling, a reversible epigenetic process[27–28], suggesting not only treatment possibilities but also cure potential. While solid tissue modification remains challenging, blood tissue modification, particularly in immune cells, is feasible[29]. As tissue enrichment analysis confirmed, immune cells and tissues are crucial in the progression of GSA-associated diseases, further supporting the potential for curing GSA-associated diseases. In fact, CAR-T has already been used in the treatment of SLE[30]. Module Three represents the JAK-STAT pathway, vital for immunity and inflammation[31], with nearly half of the pleiotropic genes linked to available drugs. Among these, 11 genes were druggable for skin or GIT diseases. Notably, Adalimumab, Infliximab, Ustekinumab, and Risankizumab can simultaneously treat skin and GIT diseases, targeting *TNF* and *IL-12B*, both of which are in this pathway. The central genes of the *JAK* family, including *JAK1*, *JAK2*, *JAK3*, and *TYK2*, are also important drug targets for GSA-associated diseases[31]. Previous studies mainly focused on *JAK1*, *JAK2*, and *JAK3*[32]. While providing therapeutic effects, they also brought obvious side effects[32]. Nowadays, *TYK2* has gained attention with fewer target organs[32]. Whether there are drug targets in the JAK-STAT pathway that only work on skin and GIT diseases (such as Vedolizumab targeting α4β7 integrin, which is only expressed in GIT tissues[33]) remains to be explored. To summarize, the JAK-STAT pathway is essential for understanding the comorbidity of skin and GIT diseases, as well as for drug repurposing and discovery. Module Four involves the antigen processing and presentation mediated by HLA genes, reflecting the dual roles of skin and GIT tissues in xenobiotic defense and metabolic exchange[34–35].

Among the five significant causal associations identified, four involved IBD, further highlighting its pivotal role in the GSA comorbidity. While previous studies have reported causal associations between skin and GIT diseases, findings regarding AD and IBD are inconsistent[36–39]. Using more recent GWAS data, we found a robust effect from IBD to AD (odds ratio [OR] = 1.067, $P = 1.36 \times 10^{-6}$). Notably, we also identified a causal effect from acne to PUD (OR = 1.129, $P = 5.20 \times 10^{-4}$), a relationship that has never been reported, and the effect direction is consistent with a recent population-based observational study[40].

Considering the importance of microbiota for GIT health, two-stage Mendelian randomization (MR) and mediation analyses were subsequently performed, revealing three potential novel biological mechanisms, including *F_Rikenellaceae*-IBD-psoriasis, *Ruminococcaceae*-IBD-psoriasis, and *Prevotella*-IBD-rosacea. Notably, *Rikenellaceae* showed potential protective effects on both skin and GIT diseases. This bacterium produces butyrate, crucial for both nutrition and protection of intestinal mucosa[41]. Riazati *et al*[42] reported that *Rikenellaceae* was negatively associated with T cell activation and blood lymphocyte concentration. Reduced *Rikenellaceae* abundance in IBD patients correlates with IBD severity[41,43]. We speculated that the reduction of *Rikenellaceae* led to

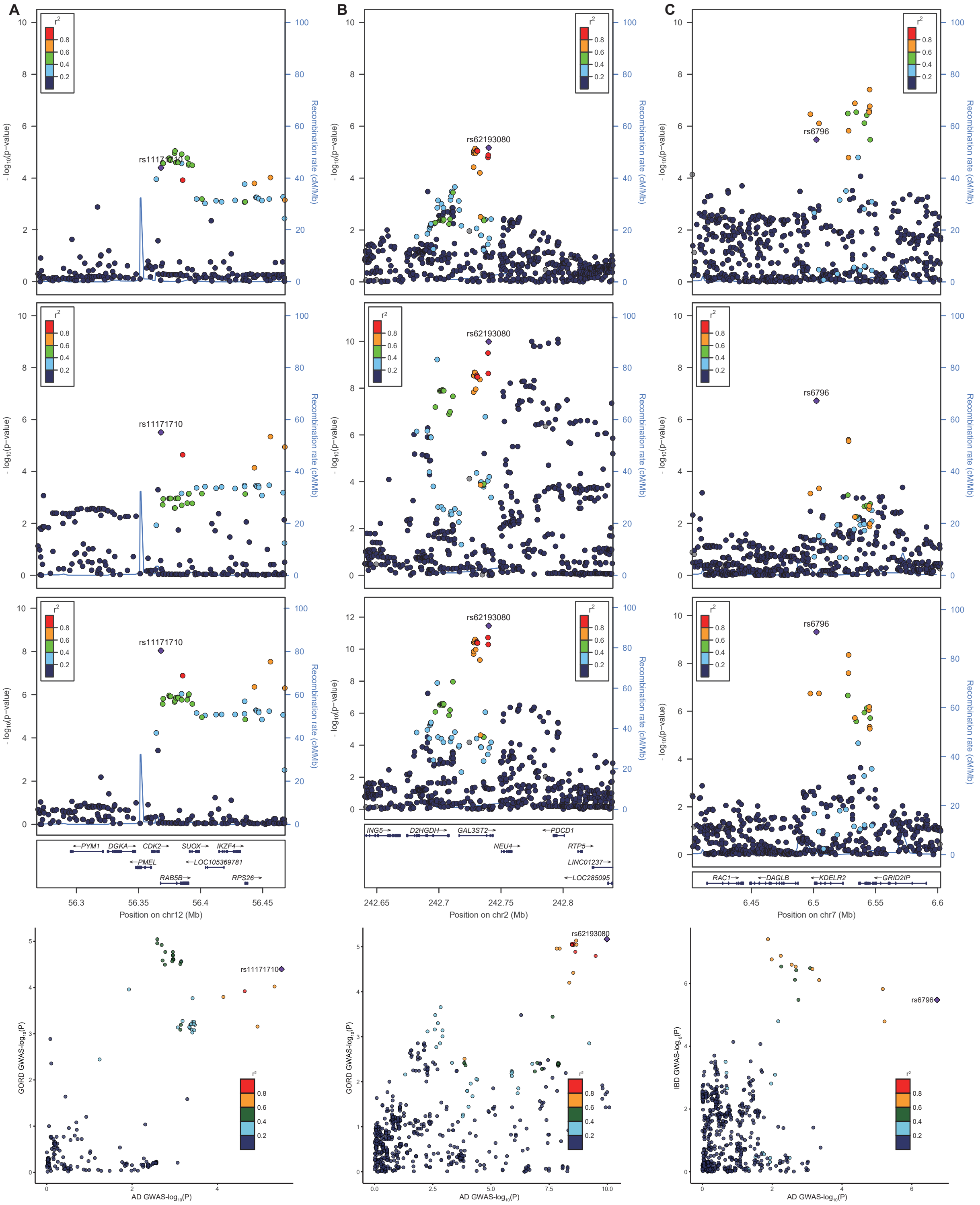
A
B
C
rs11171710
rs62193080
rs6796
r²
0.8
0.6
0.4
0.2
- log10(p-value)
Recombination rate (cM/Mb)
PYM1
DGKA
CDK2
SUOX
IKZF4
PMEL
LOC105369781
RAB5B
RPS26
Position on chr12 (Mb)
ING5
D2HGDH
GAL3ST2
PDCD1
NEU4
RTP5
LINC01237
LOC285095
Position on chr2 (Mb)
RAC1
DAGLB
KDELR2
GRID2IP
Position on chr7 (Mb)
GORD GWAS-log10(P)
AD GWAS-log10(P)
IBD GWAS-log10(P)

(Continued)

(Continued)

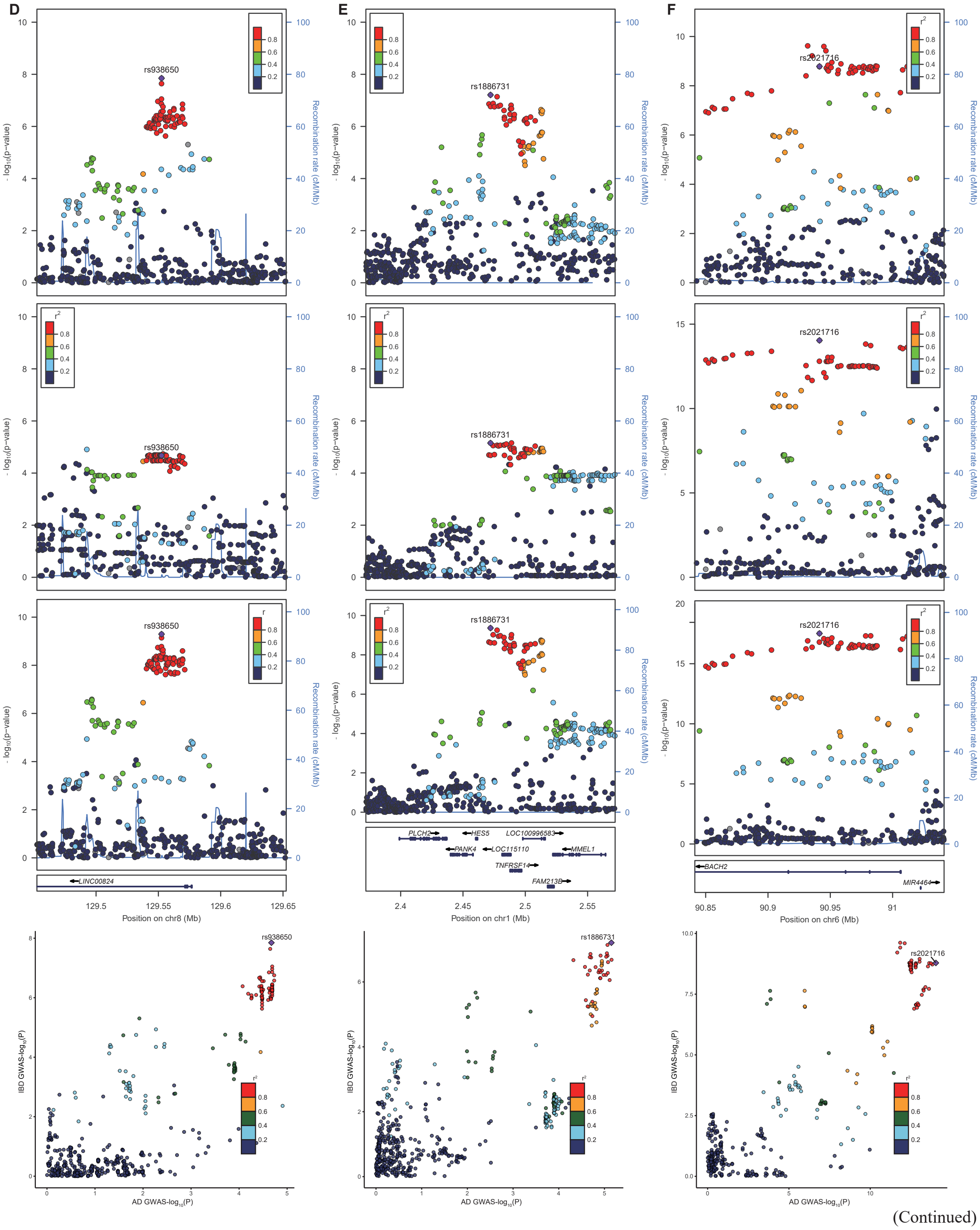

(Continued)

(Continued)

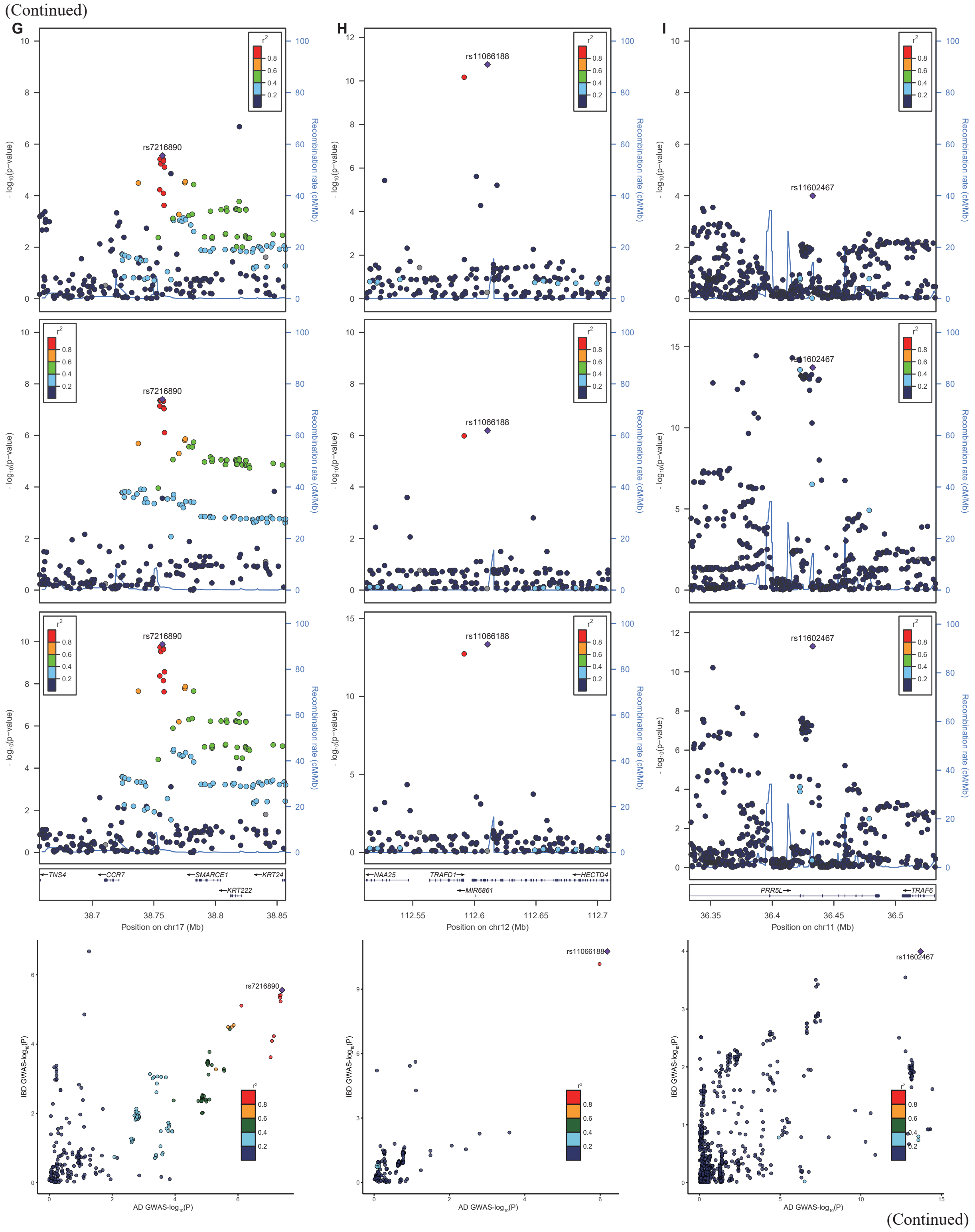

(Continued)

(Continued)

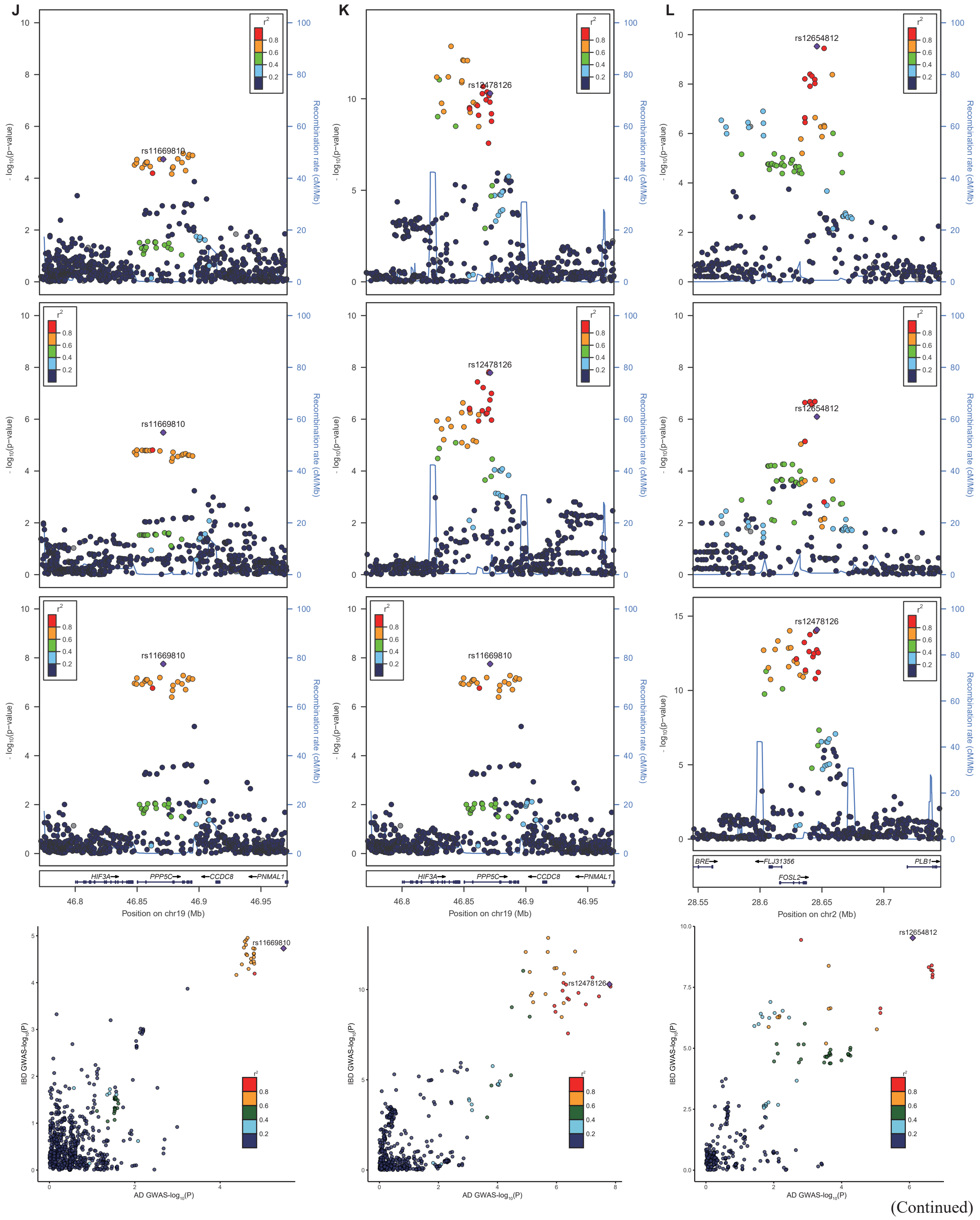

(Continued)

(Continued)

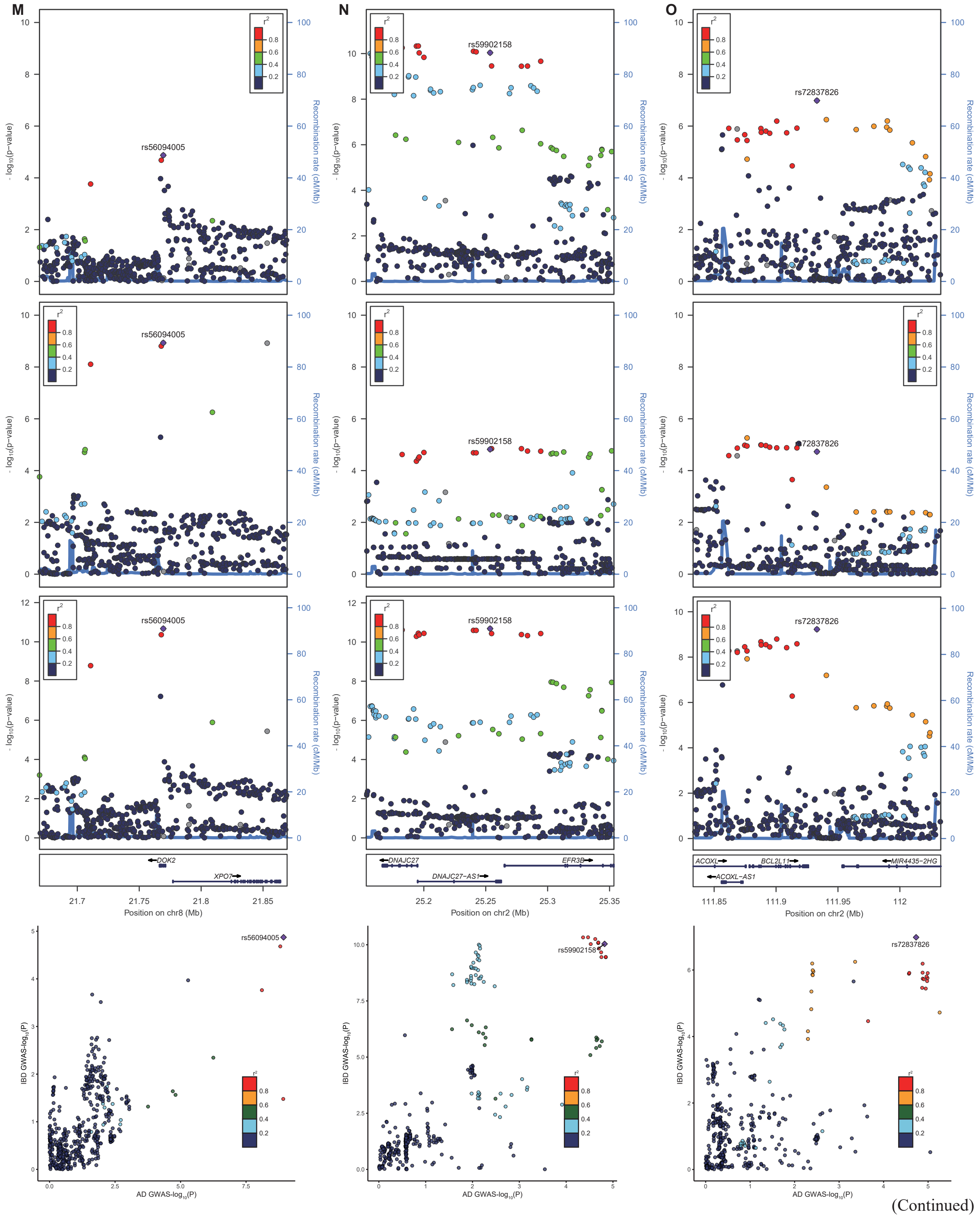

(Continued)

(Continued)

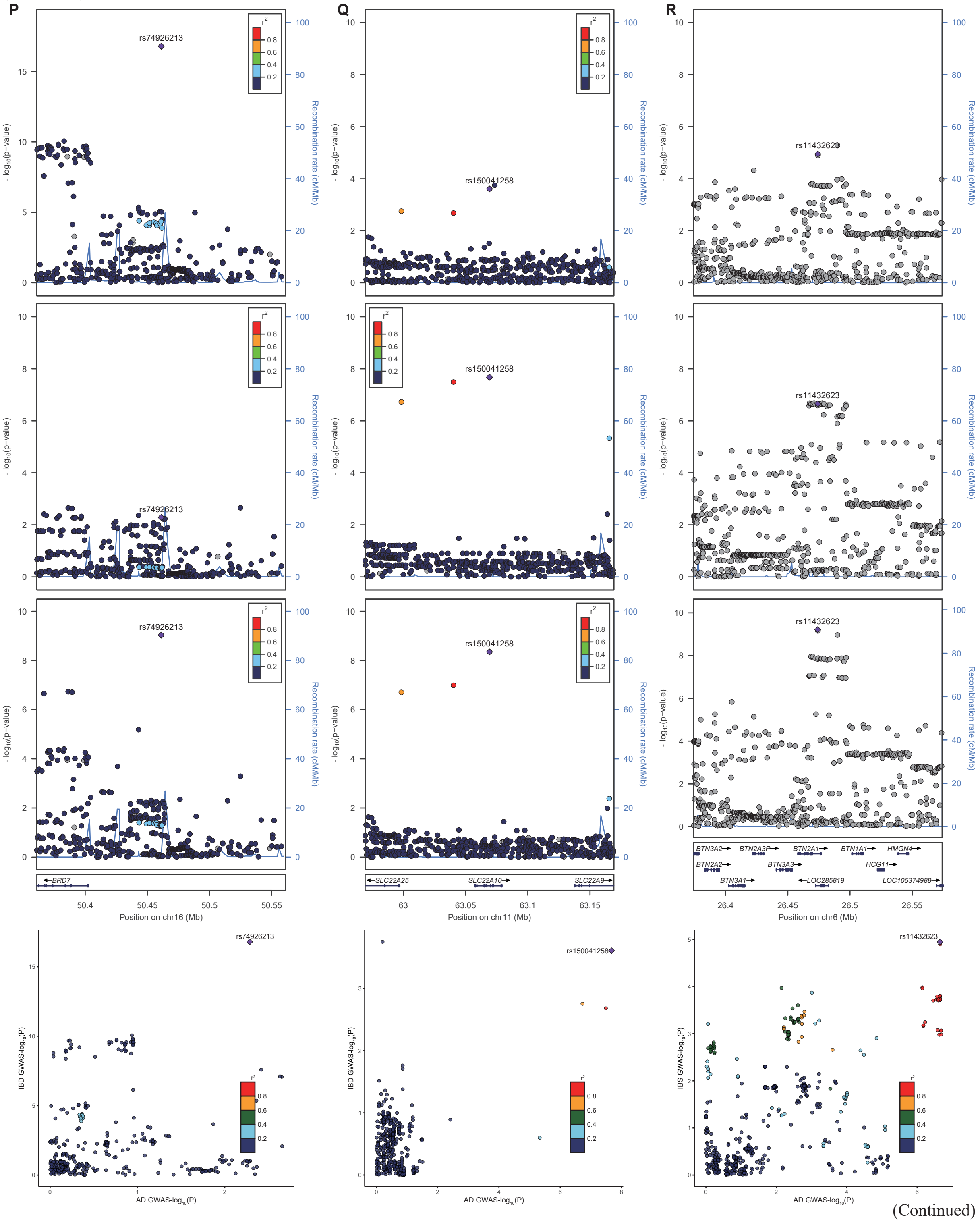

(Continued)

(Continued)

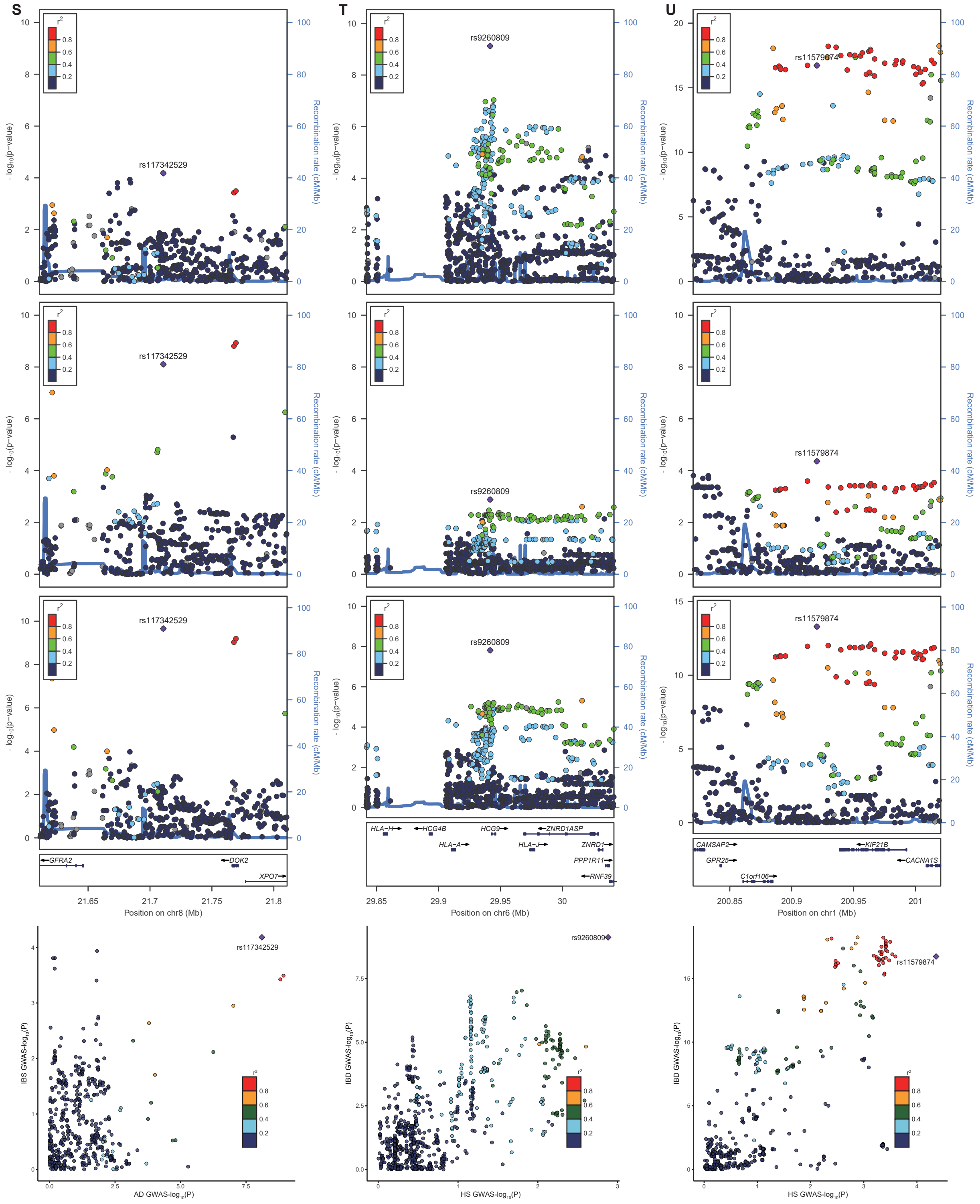

(Continued)

(Continued)

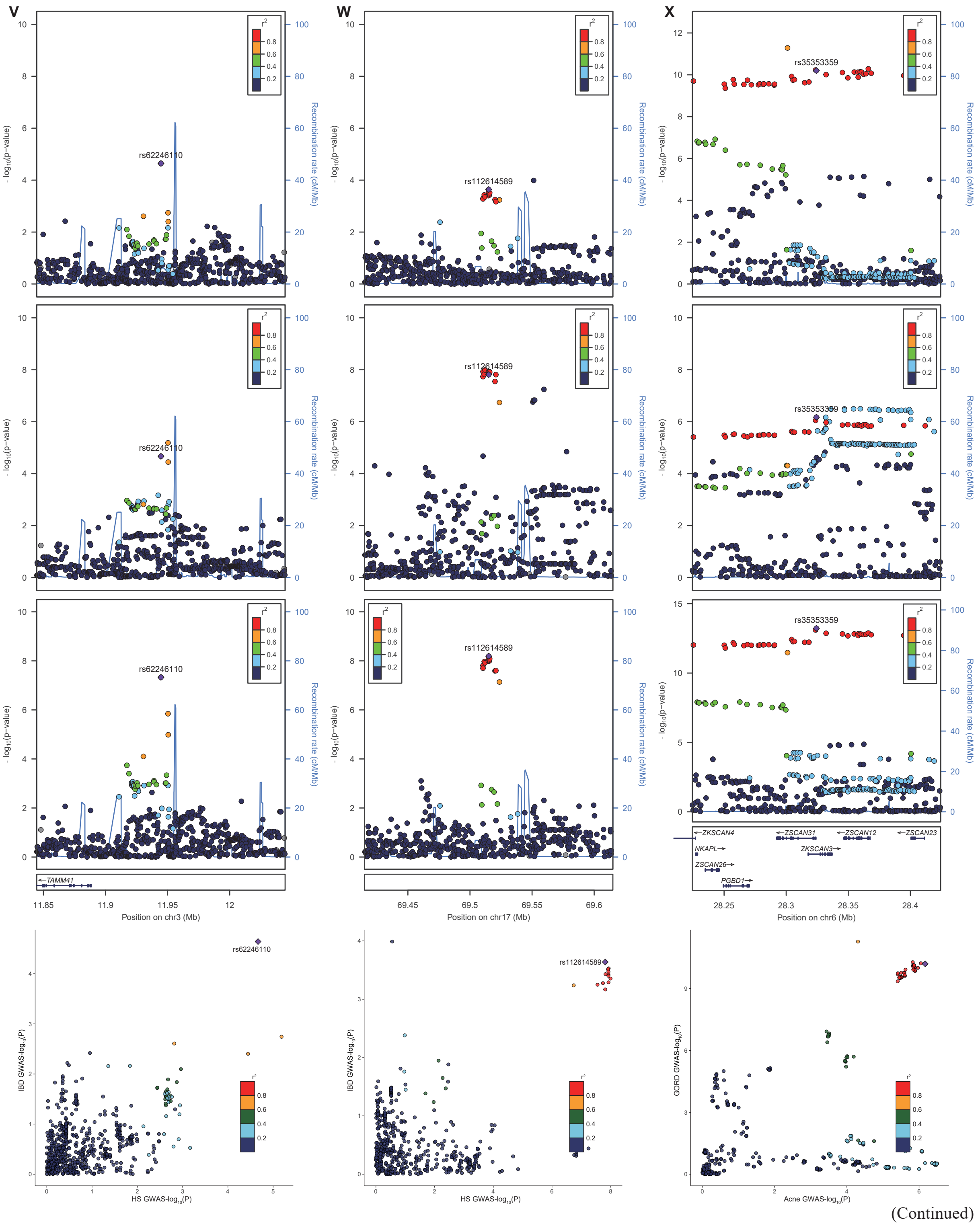

(Continued)

(Continued)

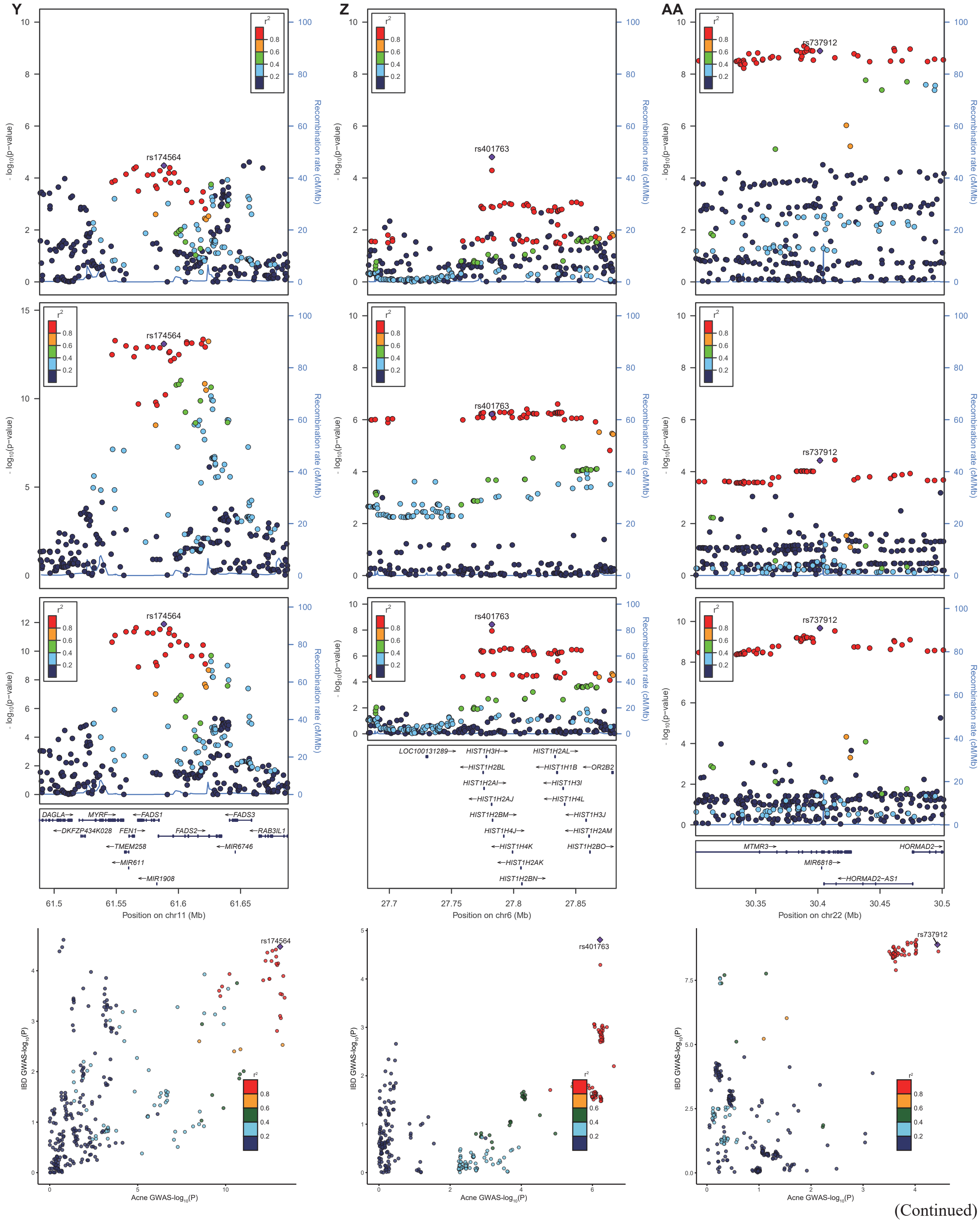

(Continued)

(Continued)

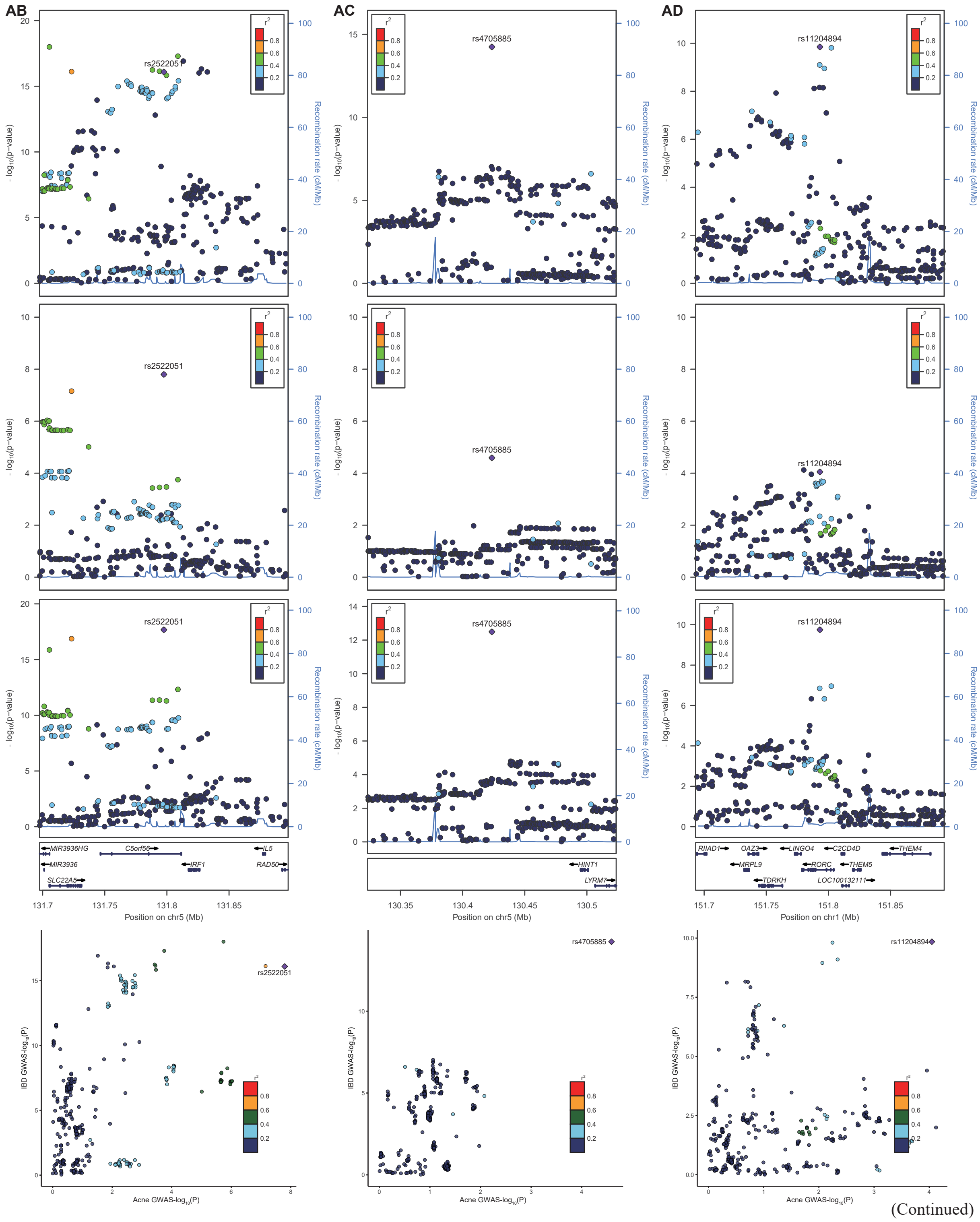

(Continued)

(Continued)

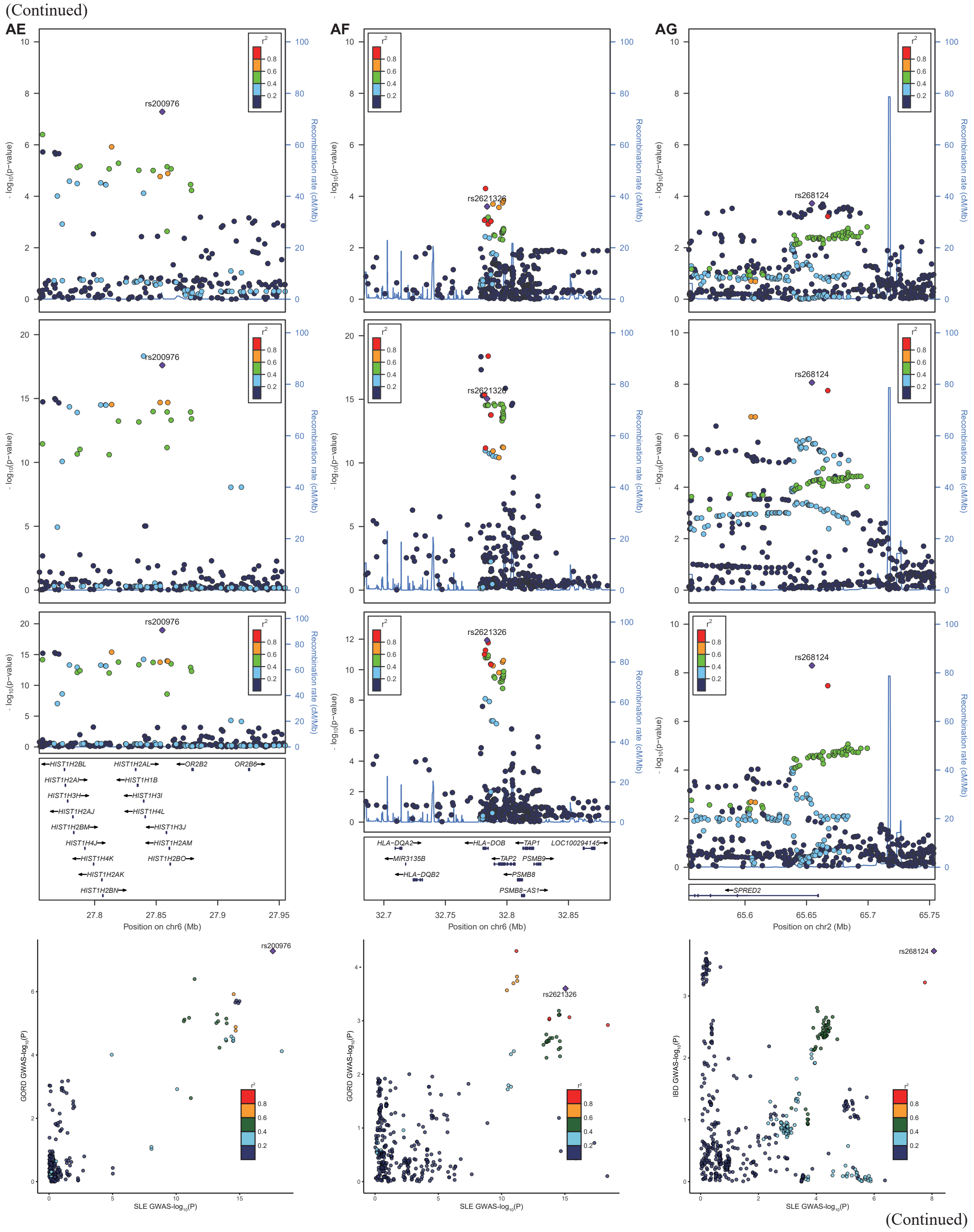

(Continued)

(Continued)

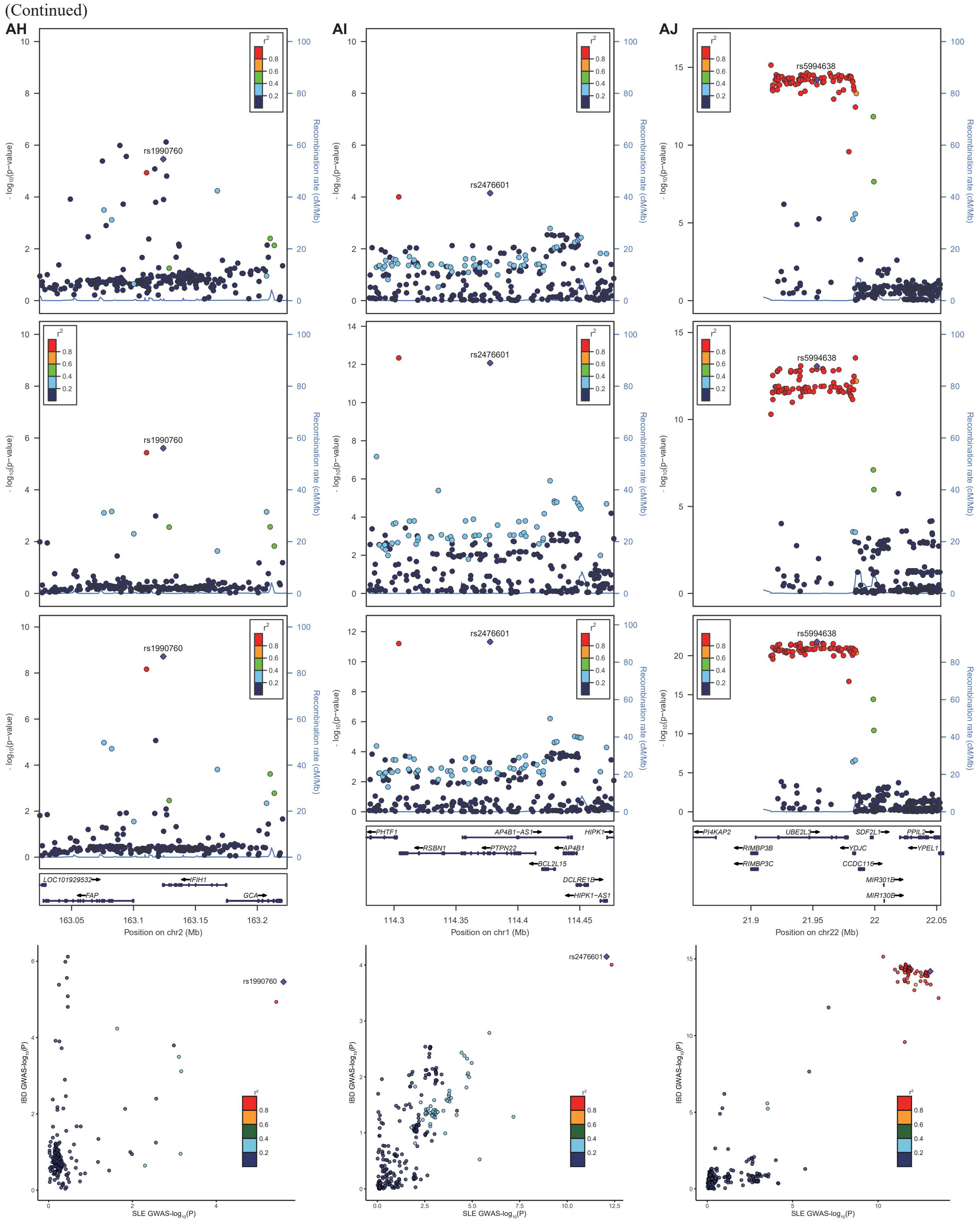

(Continued)

(Continued)

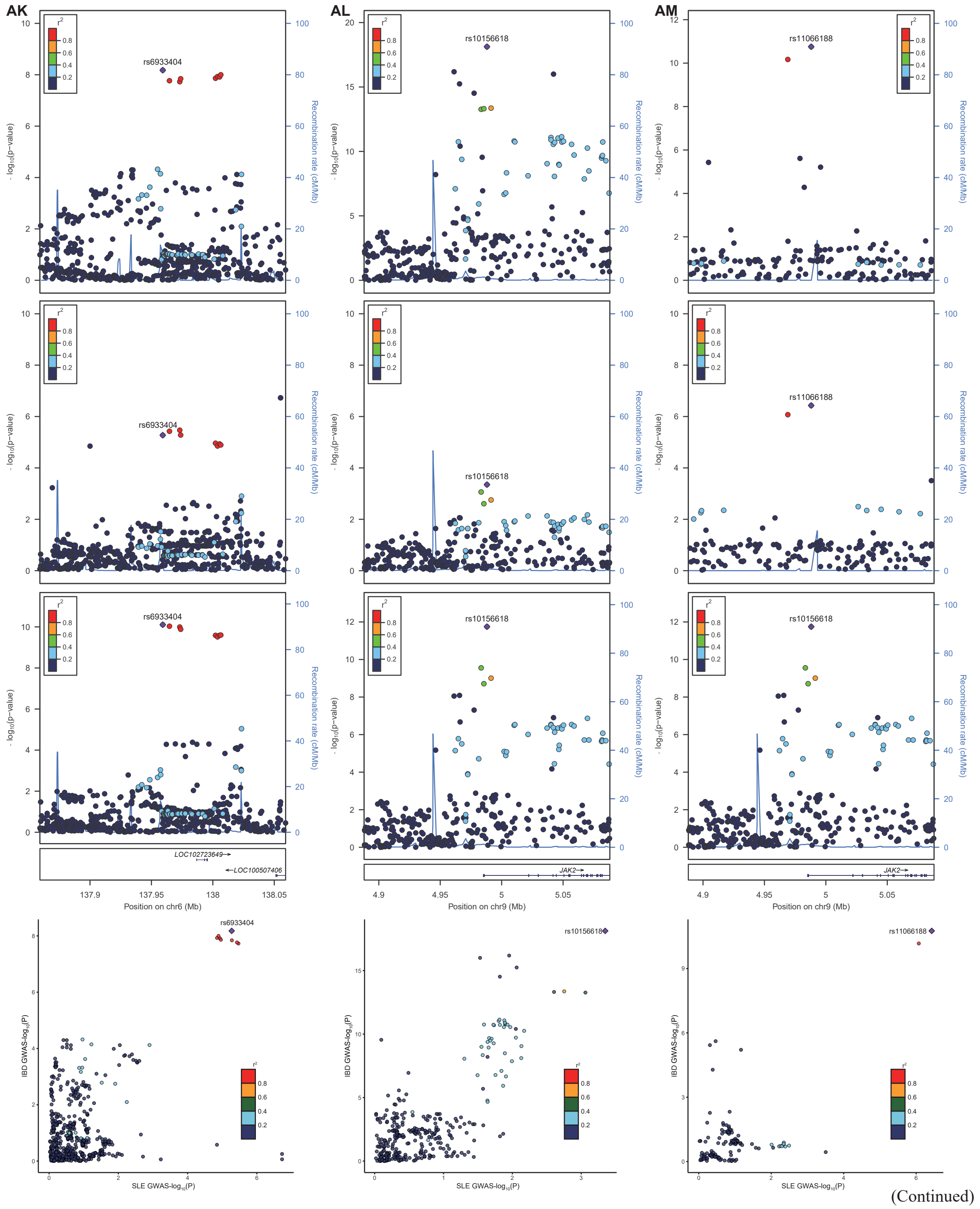

(Continued)

(Continued)

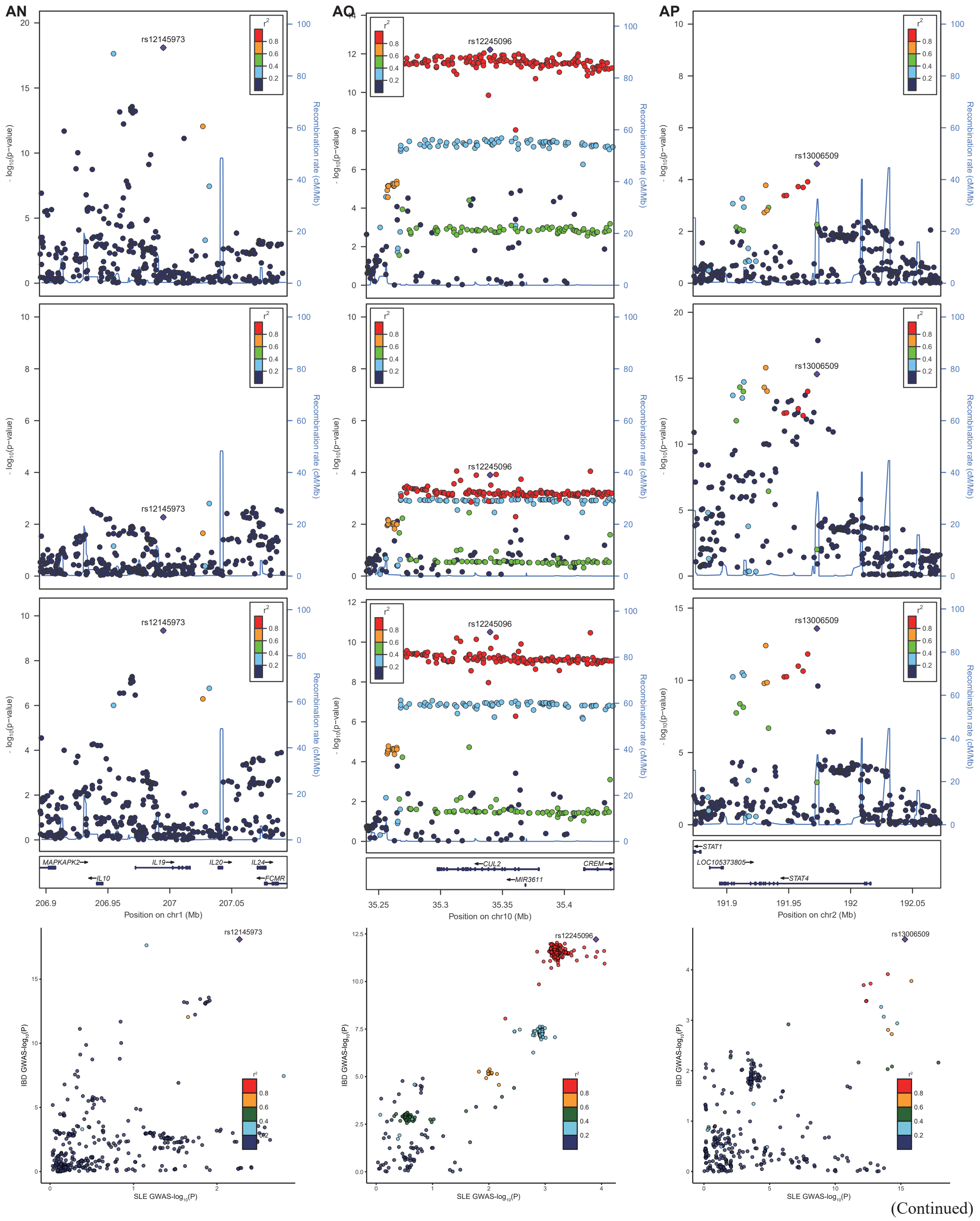

(Continued)

(Continued)

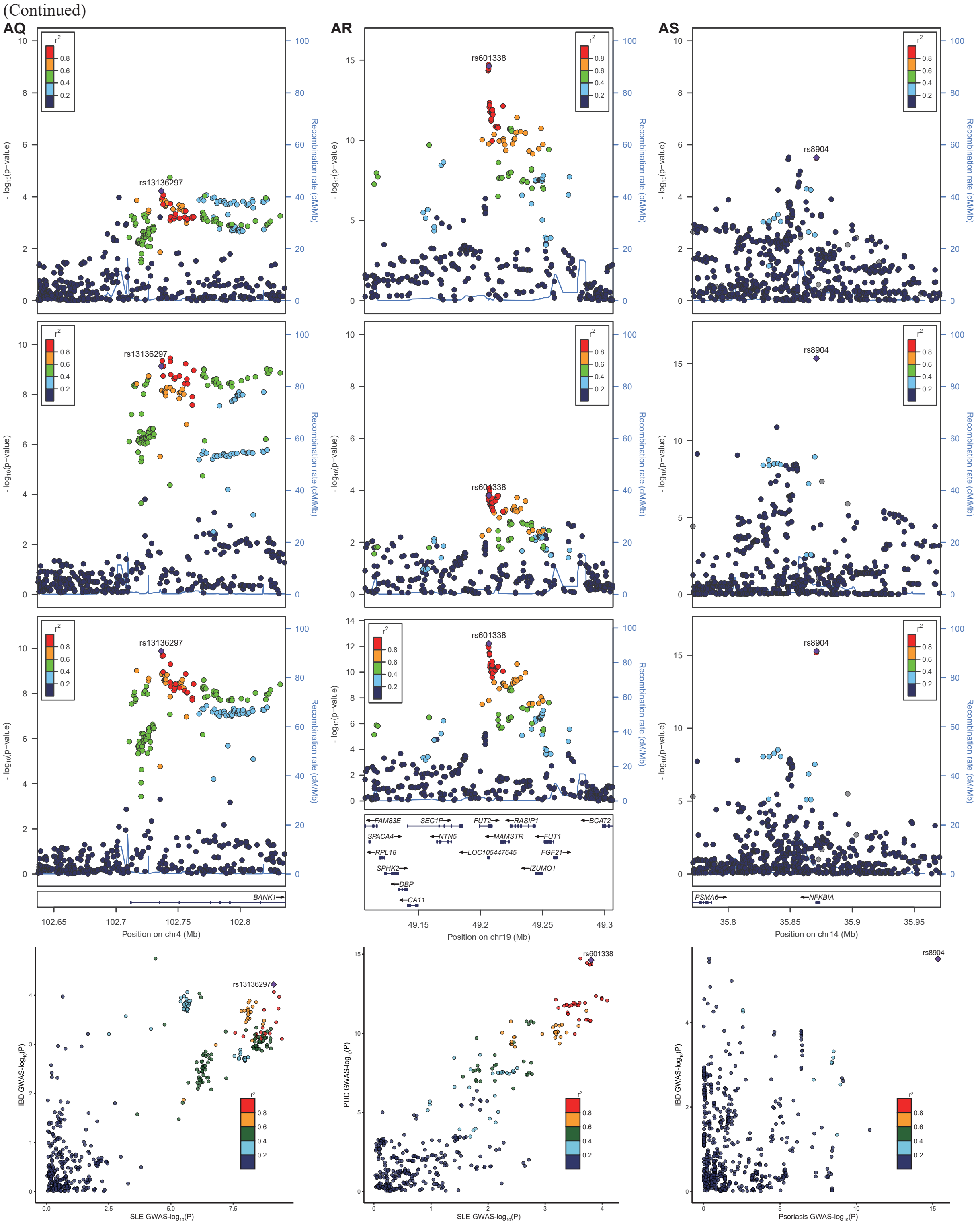

(Continued)

(Continued)

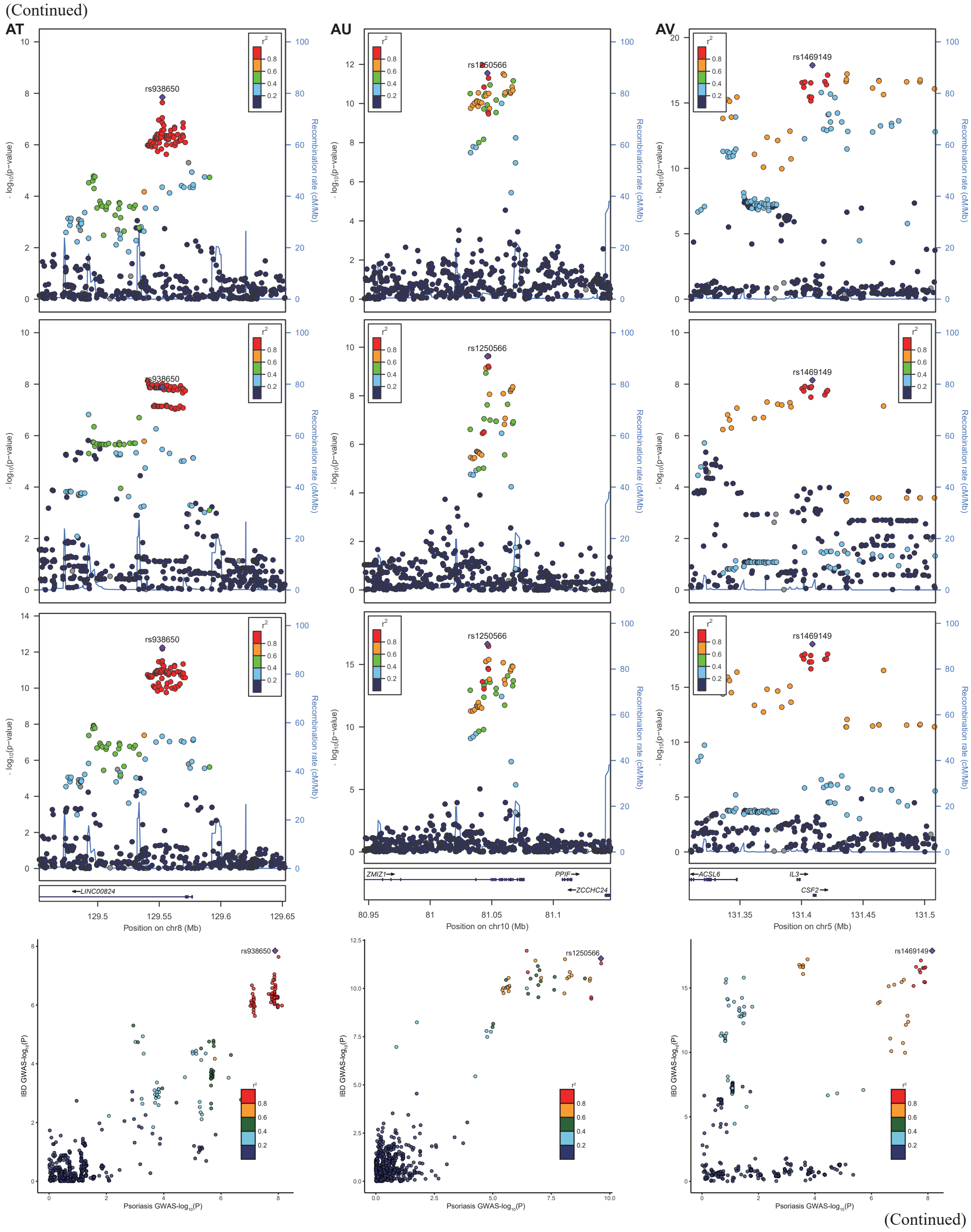

(Continued)

(Continued)

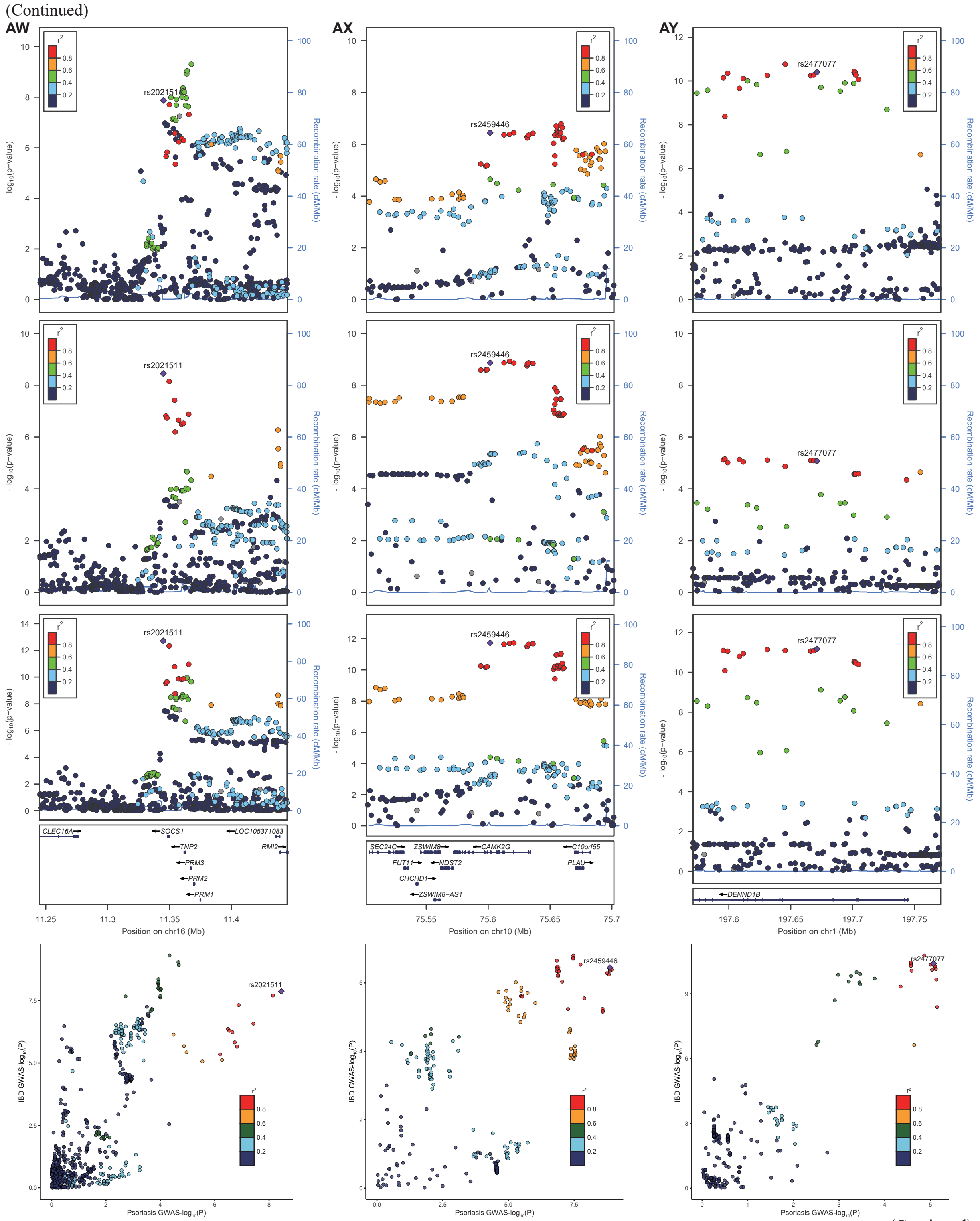

(Continued)

(Continued)

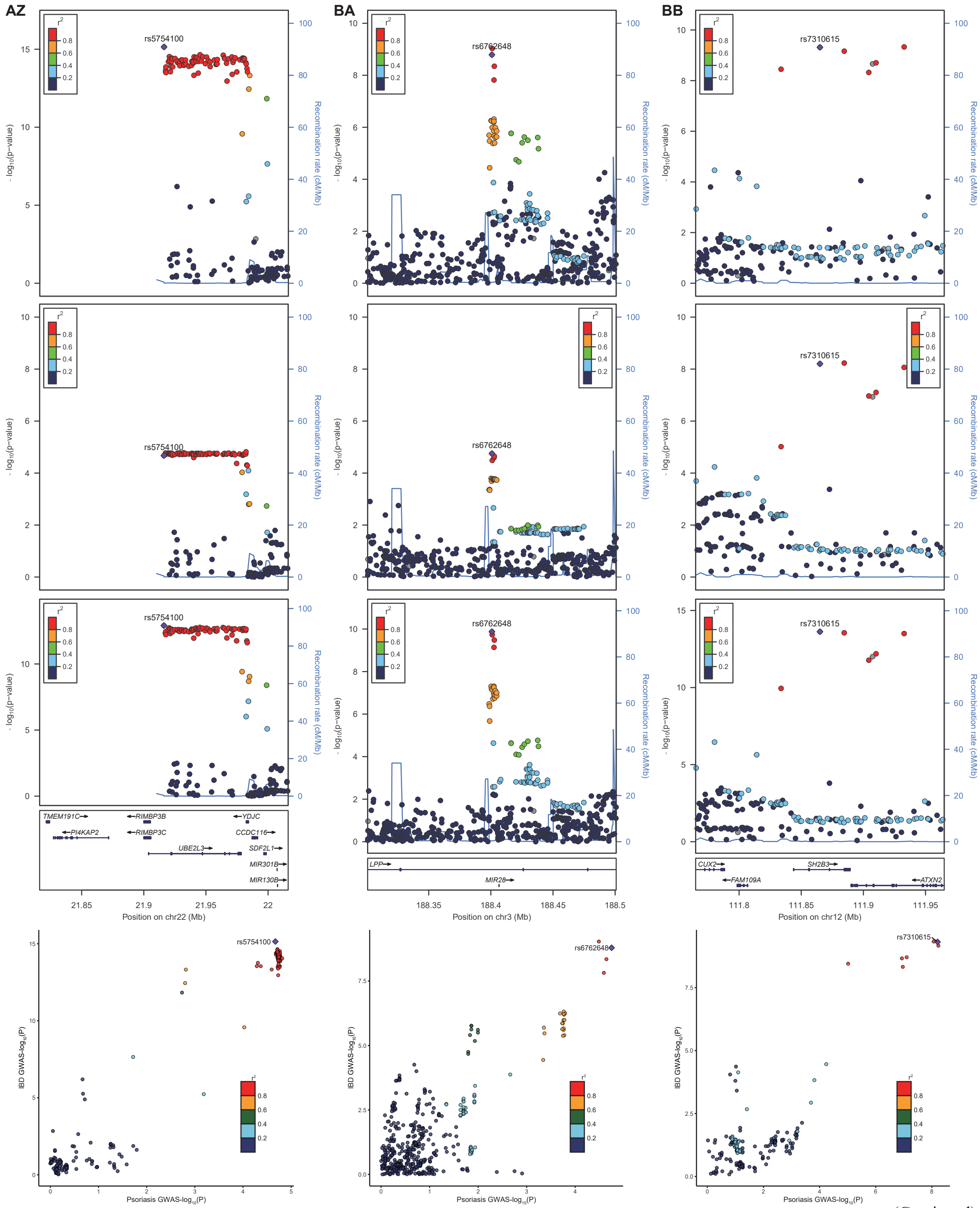

(Continued)

(Continued)

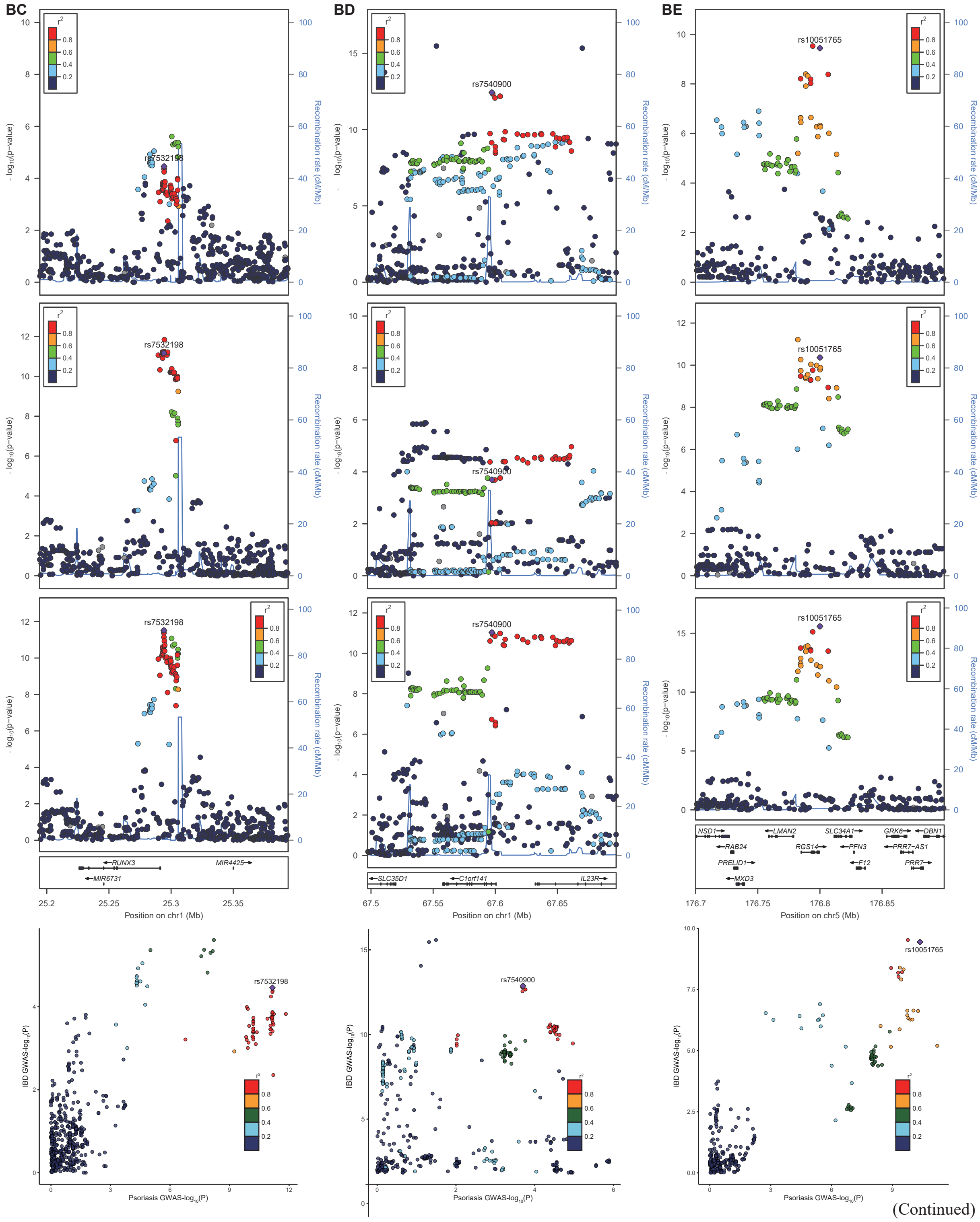

(Continued)

(Continued)

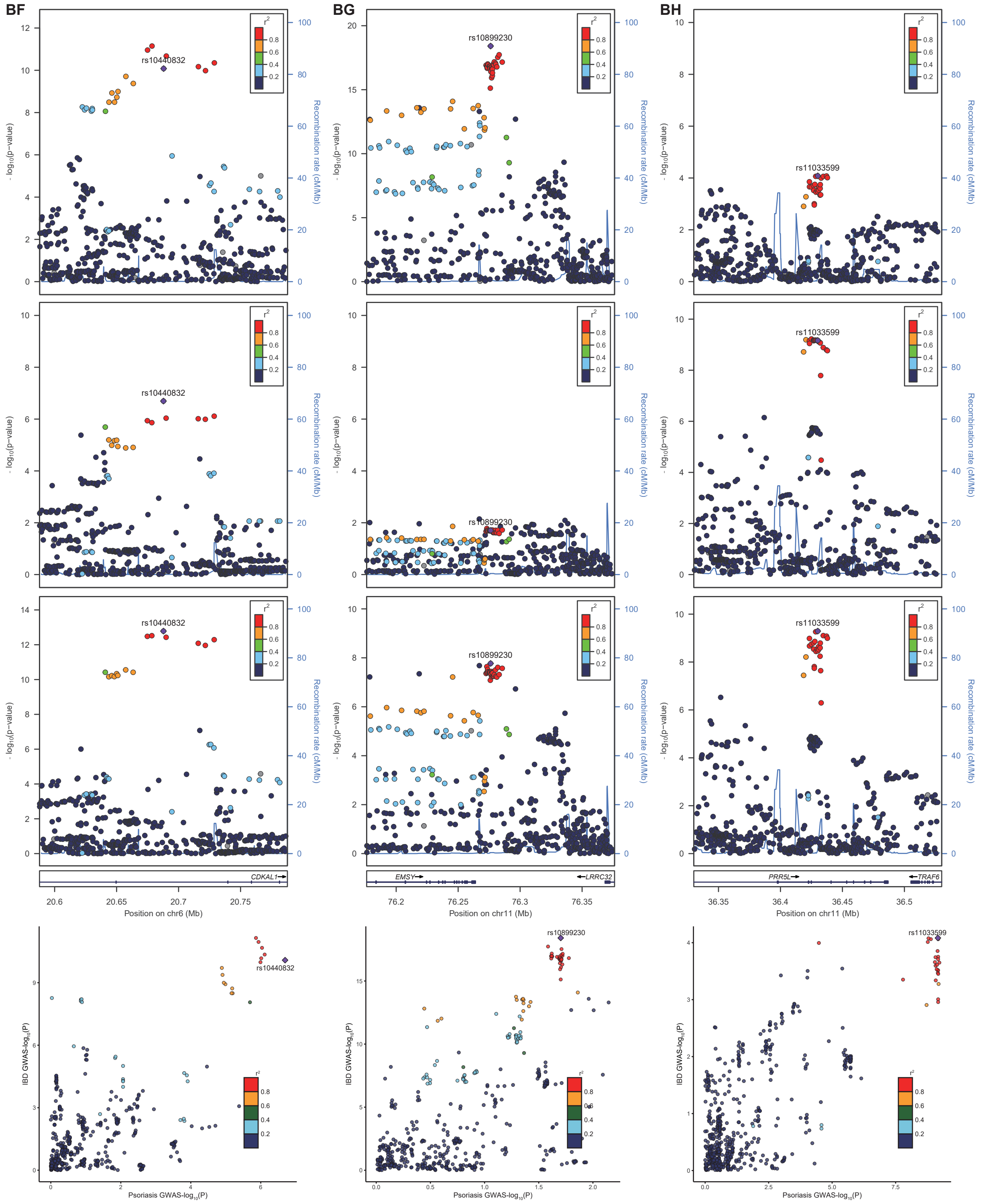

(Continued)

(Continued)

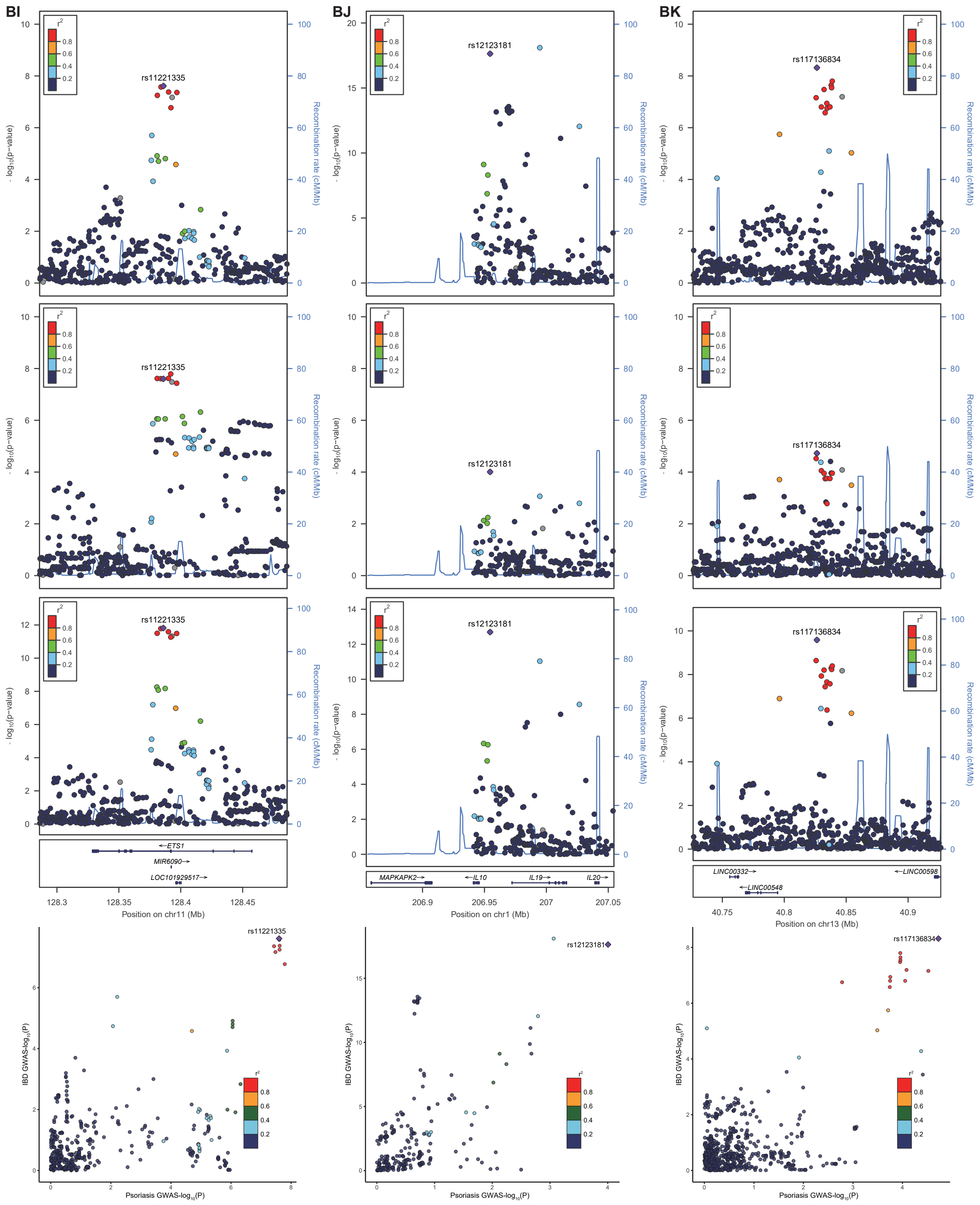

(Continued)

(Continued)

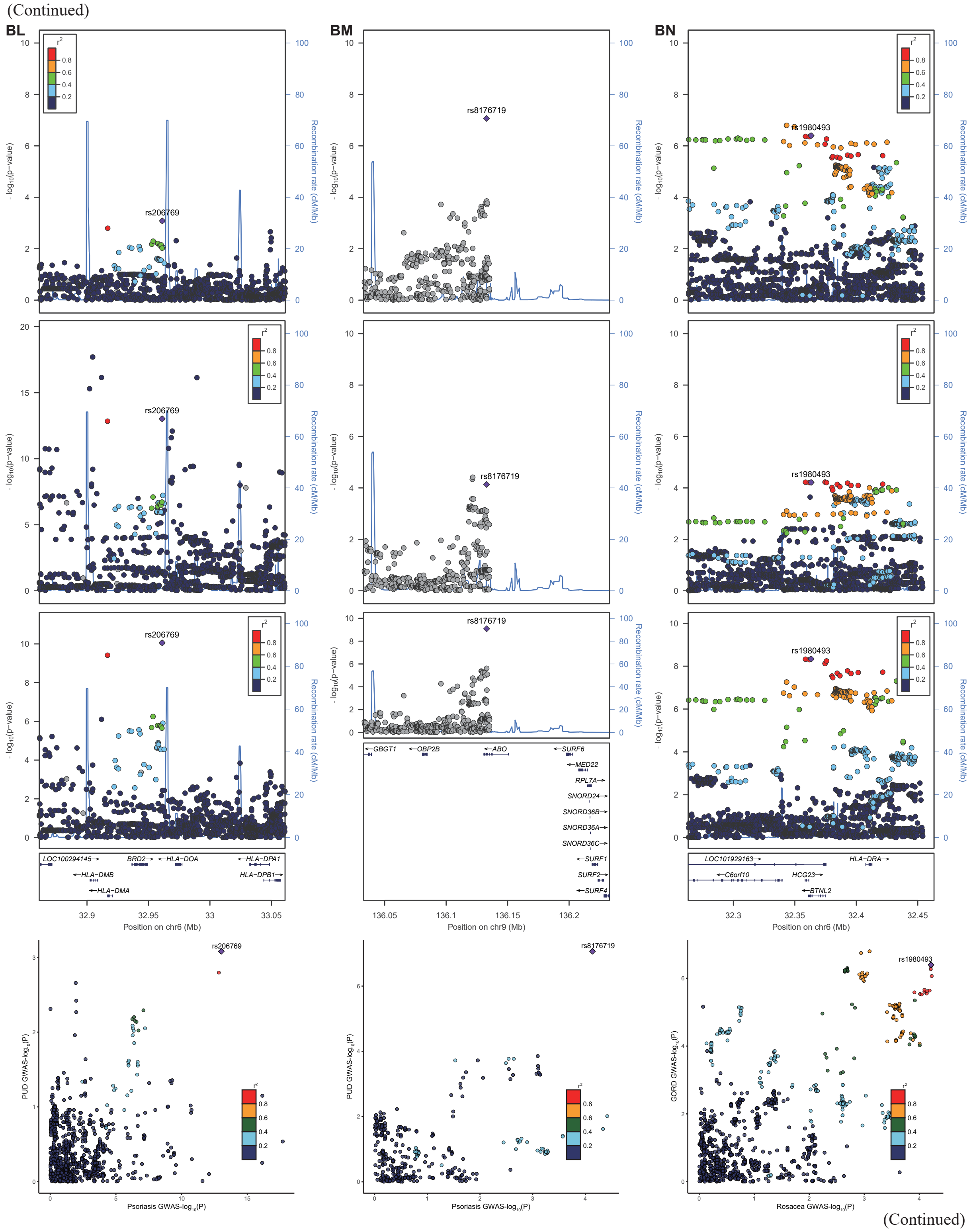

(Continued)

(Continued)

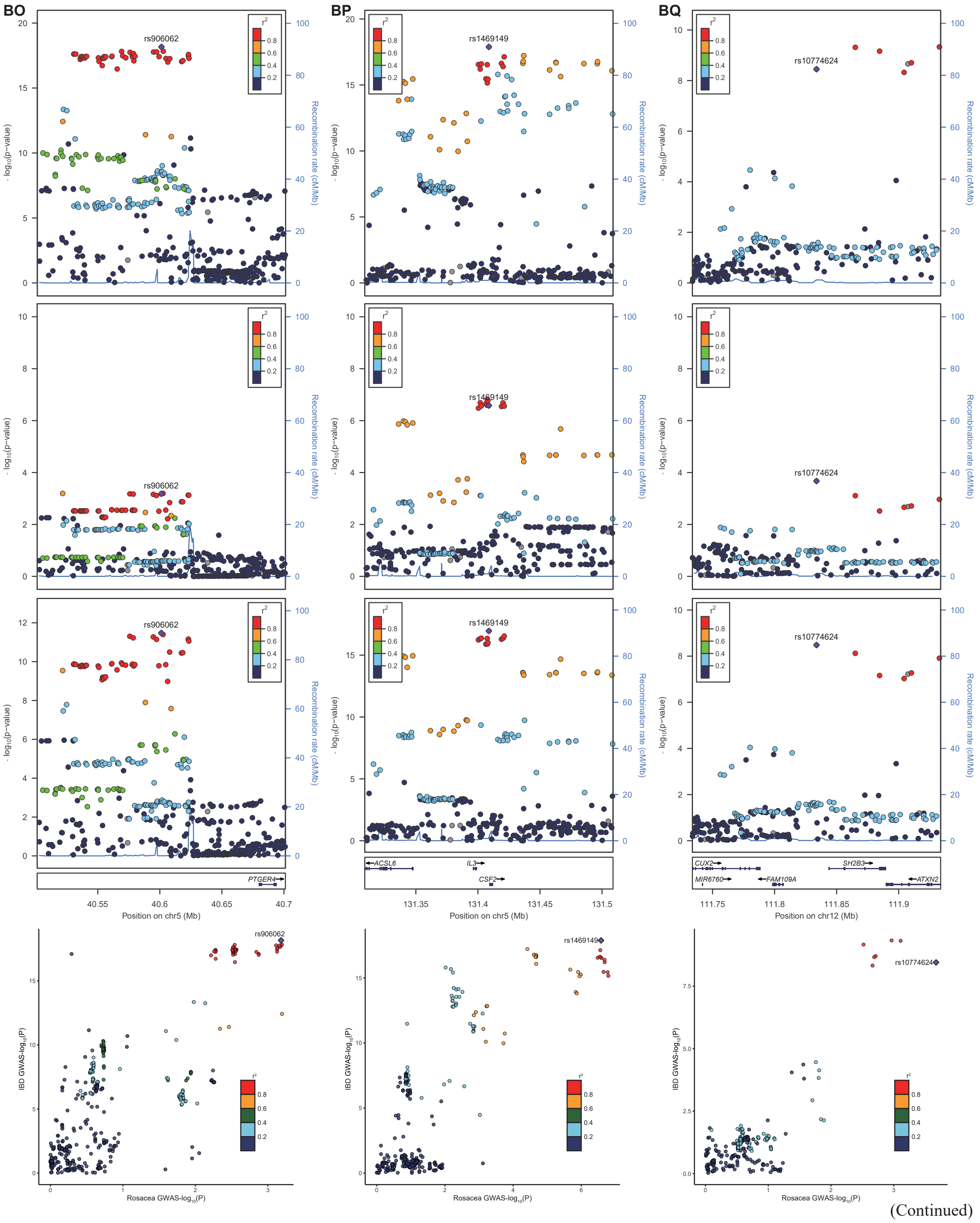

(Continued)

(Continued)

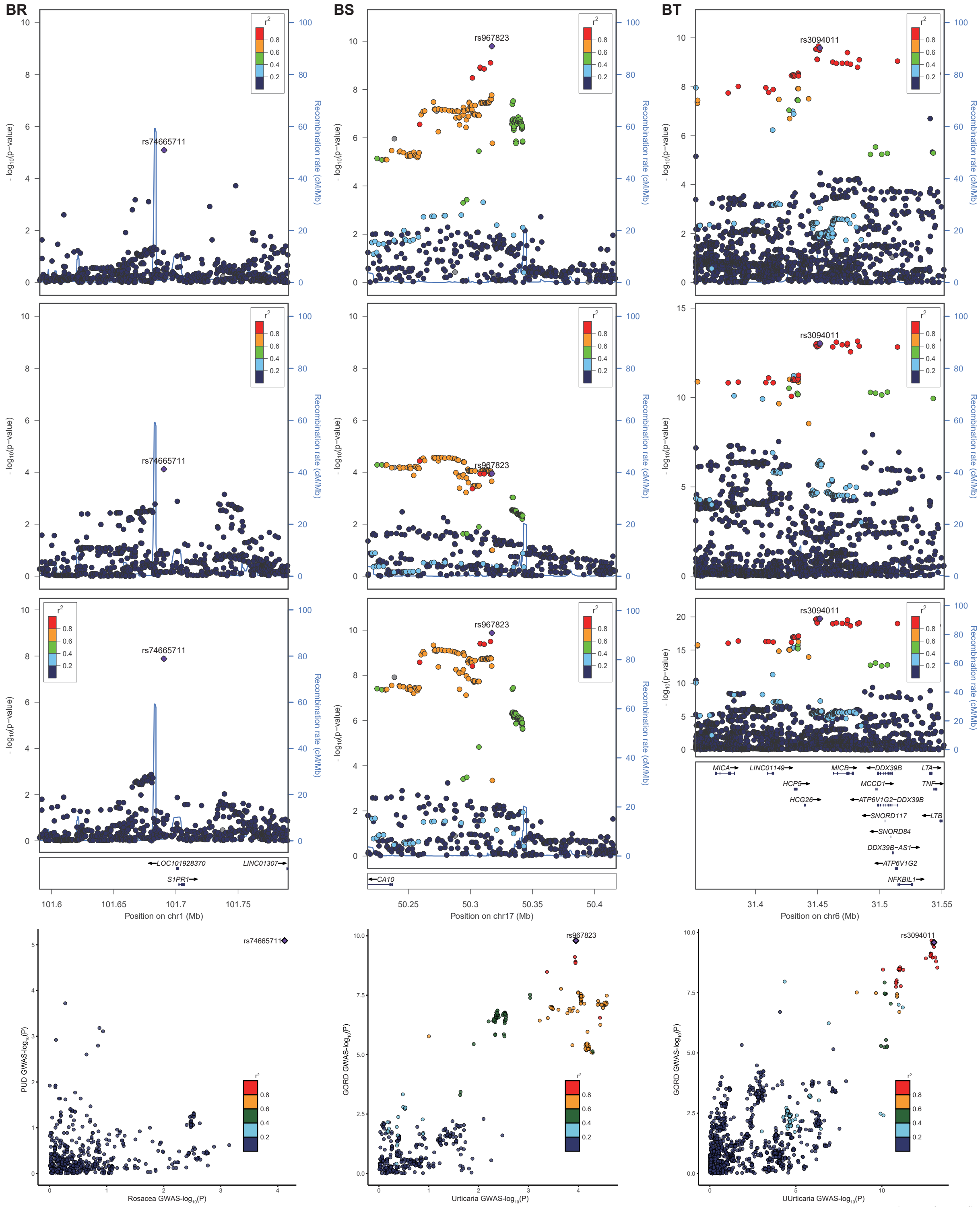

(Continued)

(Continued)

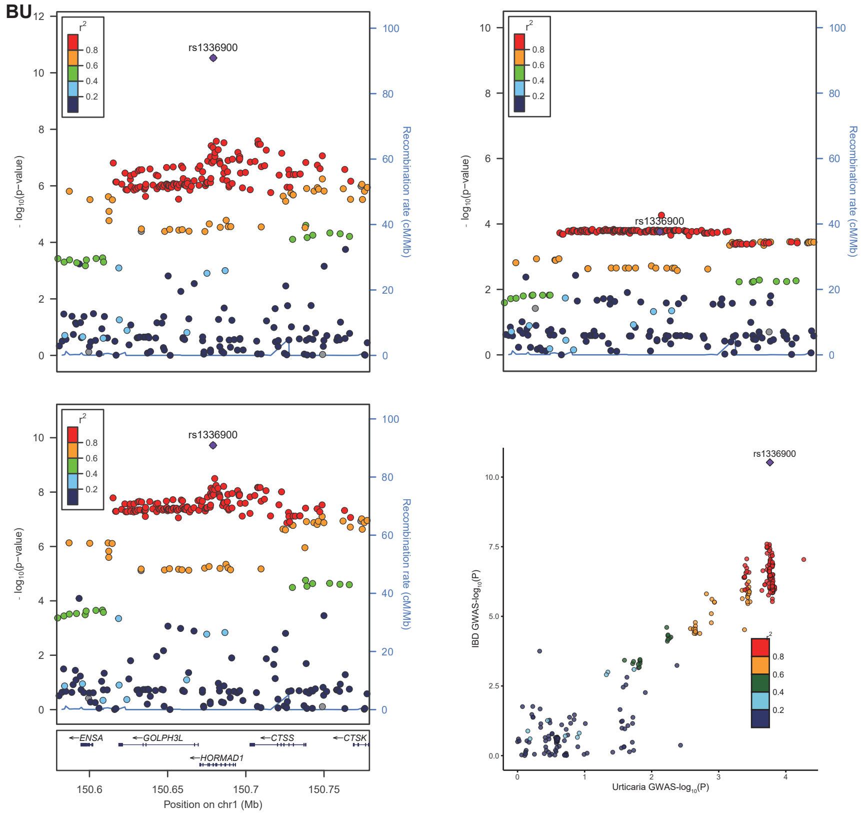

Supplementary Fig. 4 **Regional plots for colocalized loci.** A: rs11171710 (*RAB5B*, 12q13.2) in AD-GORD. B: rs62193080 (*GAL3ST2*, 2q37.3) in AD-GORD. C: rs6796 (*KDELR2*, 7p22.1) in AD-IBD. D: rs938650 (*LINC00824*, 8q24.21) in AD-IBD. E: rs1886731 (*TNFRSF14-AS1*, 1p36.32) in AD-IBD. F: rs2021716 (*BACH2*, 6q15) in AD-IBD. G: rs7216890 (*SMARCE1*, 17q21.2) in AD-IBD. H: rs11066188 (*HECTD4*, 12q24.13) in AD-IBD. I: rs11602467 (*PRR5L*, 11p12) in AD-IBD. J: rs11669810 (*PPP5C*, 19q13.32) in AD-IBD. K: rs12478126 (*FOSL2*, 2p23.2) in AD-IBD. L: rs12654812 (*RGS14*, 5q35.3) in AD-IBD. M: rs56094005 (*DOK2*, 8p21.3) in AD-IBD. N: rs59902158 (*DNAJC27-AS1*, 2p23.3) in AD-IBD. O: rs72837826 (*BCL2L11*, 2q13) in AD-IBD. P: rs74926213 (*LINC02178*, 16q12.1) in AD-IBD. Q: rs150041258 (*SLC22A10*, 11q12.3) in AD-IBD. R: rs11432623 (*LOC285819*, 6p22.2) in AD-IBS. S: rs117342529 (*DOK2*, 8p21.3) in AD-IBS. T: rs9260809 (*HCG9*, 6p22.1) in HS-IBD. U: rs11579874 (*MROH3P*, 1q32.1) in HS-IBD. V: rs62246110 (*TAMM41*, 3p25.2) in HS-IBD. W: rs112614589 (*CASC17*, 17q24.3) in HS-IBD. X: rs35353359 (*ZKSCAN3*, 6p22.1) in Acne-GORD. Y: rs174564 (*FADS2*, 11q12.2) in Acne-IBD. Z: rs401763 (*H2AC14*, *H2BC14*, 6p22.1) in Acne-IBD. AA: rs737912 (*MTMR3*, 22q12.2) in Acne-IBD. AB: rs2522051 (*IRF1-AS1*, 5q31.1) in Acne-IBD. AC: rs4705885 (*HINT1*, 5q23.3) in Acne-IBD. AD: rs11204894 (*RORC*, 1q21.3) in Acne-IBD. AE: rs200976 (*H3C12*, 6p22.1) in SLE-GORD. AF: rs2621326 (*HLA-DOB*, 6p21.32) in SLE-GORD. AG: rs268124 (*SPRED2*, 2p14) in SLE-IBD. AH: rs1990760 (*IFIH1*, 2q24.2) in SLE-IBD. AI: rs2476601 (*PTPN22*, 1p13.2) in SLE-IBD. AJ: rs5994638 (*UBE2L3*, 22q11.21) in SLE-IBD. AK: rs6933404 (*LOC102723649*, 6q23.3) in SLE-IBD. AL: rs10156618 (*JAK2*, 9p24.1) in SLE-IBD. AM: rs11066188 (*HECTD4*, 12q24.13) in SLE-IBD. AN: rs12145973 (*IL19*, 1q32.1) in SLE-IBD. AO: rs12245096 (*CUL2*, 10p11.21) in SLE-IBD. AP: rs13006509 (*STAT4*, 2q32.3) in SLE-IBD. AQ: rs13136297 (*BANK1*, 4q24) in SLE-IBD. AR: rs601338 (*FUT2*, 19q13.33) in SLE-PUD. AS: rs8904 (*NFKBIA*, 14q13.2) in Psoriasis-IBD. AT: rs938650 (*LINC00824*, 8q24.21) in Psoriasis-IBD. AU: rs1250566 (*ZMIZ1*, 10q22.3) in Psoriasis-IBD. AV: rs1469149 (*CSF2*, 5q31.1) in Psoriasis-IBD. AW: rs2021511 (*SOCS1*, 16p13.13) in Psoriasis-IBD. AX: rs2459446 (*CAMK2G*, 10q22.2) in Psoriasis-IBD. AY: rs2477077 (*DENND1B*, 1q31.3) in Psoriasis-IBD. AZ: rs5754100 (*UBE2L3*, 22q11.21) in Psoriasis-IBD. BA: rs6762648 (*LPP*, 3q28) in Psoriasis-IBD. BB: rs7310615 (*SH2B3*, 12q24.12) in Psoriasis-IBD. BC: rs7532198 (*RUNX3*, 1p36.11) in Psoriasis-IBD. BD: rs7540900 (*C1orf141*, 1p31.3) in Psoriasis-IBD. BE: rs10051412 (*IRGM*, 5q33.1) in Psoriasis-IBD. BF: rs10440832 (*CDKAL1*, 6p22.3) in Psoriasis-IBD. BG: rs10899230 (*EMSY*, 11q13.5) in Psoriasis-IBD. BH: rs11033599 (*PRR5L*, 11p12) in Psoriasis-IBD. BI: rs11221335 (*ETS1*, 11q24.3) in Psoriasis-IBD. BJ: rs12123181 (*IL19*, 1q32.1) in Psoriasis-IBD. BK: rs117136834 (*LINC00548*, 13q14.11) in Psoriasis-IBD. BL: rs206769 (*HLA-DOA*, 6p21.32) in Psoriasis-PUD. BM: rs8176719 (*ABO*, 9q34.2) in Psoriasis-PUD. BN: rs1980493 (*TSBP1-AS1*, 6p21.32) in Rosacea-GORD. BO: rs906062 (*PTGER4*, 5p13.1) in Rosacea-IBD. BP: rs1469149 (*CSF2*, 5q31.1) in Rosacea-IBD. BQ: rs10774624 (*SH2B3*, 12q24.12) in Rosacea-IBD. BR: rs74665711 (*LOC101928370*, 1p21.2) in Rosacea-PUD. BS: rs967823 (*CA10*, 17q22) in Urticaria-GORD. BT: rs3094011 (*MICB-DT*, 6p21.33) in Urticaria-GORD. BU: rs1336900 (*HORMAD1*, 1q21.3) in Urticaria-IBD. From top to bottom, four panels are GIT GWAS, skin GWAS, PLACO analysis, and a comparison of the summary statistics of GIT and skin traits. The lead variants in PLACO analyses were colored purple. Other variants were colored according to their LD to the lead variants. Abbreviations: AD, atopic dermatitis; GORD, gastro-oesophageal reflux disease; HS, hidradenitis suppurativa; IBD, inflammatory bowel disease; LD, linkage disequilibrium; PUD, peptic ulcer disease; SLE, systemic lupus erythematosus.

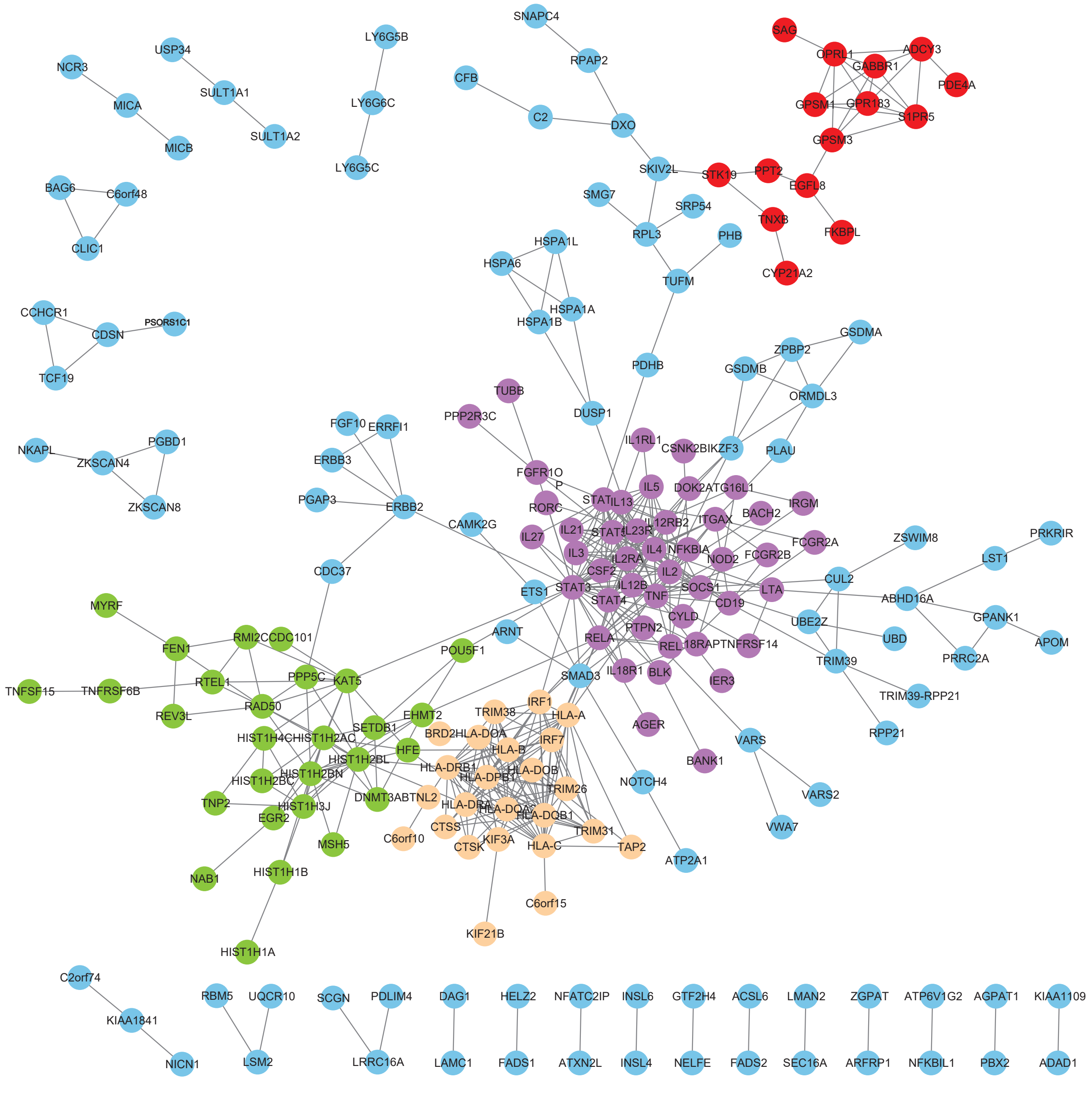

Supplementary Fig. 5 **GSA-PPIN identified with protein-protein interaction analysis.** A total of four significant modules were identified in GSA-PPIN using the Louvain module detection algorithm. Modules One to Four were colored in red, green, purple, and yellow, respectively. The reference panel was pre-defined using the STRING database. The significance of modules was determined using the qs-test.

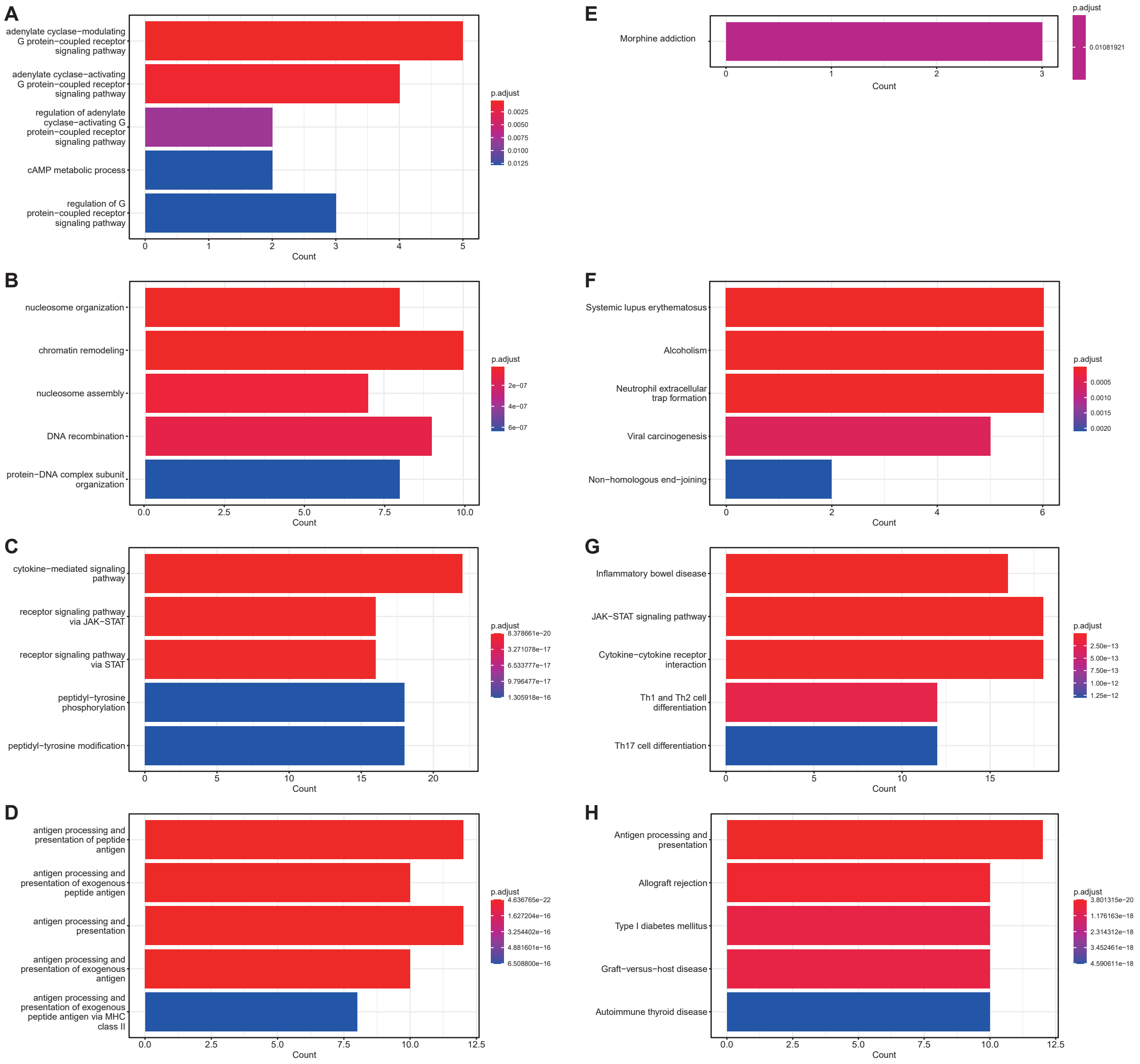

Supplementary Fig. 6 **GO and KEGG pathway enrichment for four significant PPI modules.** A–D: GO enrichment results for Module One to Module Four. E–F: KEGG enrichment results for Module One to Module Four. A and E: Module One was enriched in the G protein−coupled receptor signaling pathway and might be associated with Morphine addiction. B and F: Module Two was enriched in chromatin remodeling. C and G: Module Three might participate in the JAK-STAT signaling pathway and Th cell differentiation. D and H: Module Four was associated with antigen processing and presentation. Only the top five significant enrichment results are shown (Module One had only one significant KEGG pathway). The significance was declared at Benjamini-Hochberg (BH) adjusted $P < 0.05$. Count, number of genes in the module found in the corresponding entry. *P*.adjust is the *P* adjusted with BH correction.

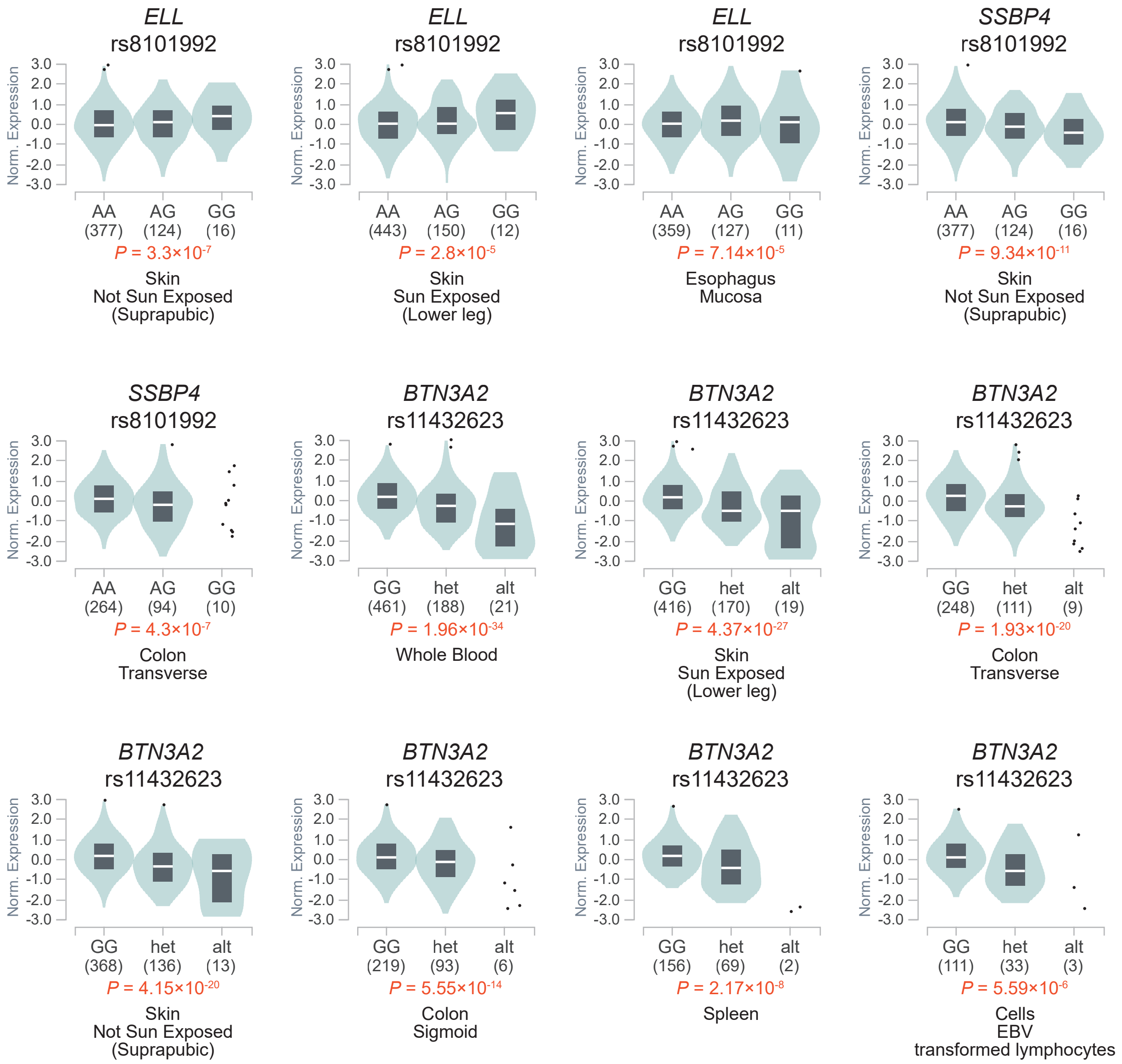

Supplementary Fig. 7 **The eQTL regulatory information of rs8101992 and rs11432623.**

the decrease in butyrate, which could disrupt intestinal mucosa homeostasis, promote T cell activation, and subsequently contribute to the development of IBD as well as psoriasis.

Supplementary Tables

Supplementary Tables 1–13 are included in the **Supplementary Tables** available at http://www.jbr-pub.org.cn/article/doi/10.7555/JBR.39.20250166?pageType=en.

References

[1] Baldaccini M, Pfeffer S. Untangling the roles of RNA helicases in antiviral innate immunity[J]. *PLoS Pathog*, 2021, 17(12): e1010072.

[2] Zulkafli NES, Majeed AY, Ad'hiah AH. A novel intergenic variant linked to IFIH1 rs1990760 polymorphism, rs2111485, shows an association with susceptibility to coronavirus disease 2019 and influences IFIH1 protein levels[EB/OL]. [2025-04-01]. https://www.authorea.com/doi/full/10.22541/au.171167073.39246049.

[3] Gorman JA, Hundhausen C, Errett JS, et al. The A946T variant of the RNA sensor IFIH1 mediates an interferon program that limits viral infection but increases the risk for autoimmunity[J]. *Nat Immunol*, 2017, 18(7): 744–752.

[4] Arnolds KL, Martin CG, Lozupone CA. Blood type and the microbiome- untangling a complex relationship with lessons from pathogens[J]. *Curr Opin Microbiol*, 2020, 56: 59–66.

[5] Wacklin P, Makivuokko H, Alakulppi N, et al. Secretor genotype (*FUT2* gene) is strongly associated with the composition of *Bifidobacteria* in the human intestine[J]. *PLoS One*, 2011, 6(5): e20113.

[6] Jostins L, Ripke S, Weersma RK, et al. Host-microbe interactions have shaped the genetic architecture of inflammatory bowel disease[J]. *Nature*, 2012, 491(7422): 119–124.

[7] Rühlemann MC, Hermes BM, Bang C, et al. Genome-wide association study in 8, 956 German individuals identifies influence of ABO histo-blood groups on gut microbiome[J]. *Nat Genet*, 2021, 53(2): 147–155.

[8] Qin Y, Havulinna AS, Liu Y, et al. Combined effects of host genetics and diet on human gut microbiota and incident disease in a single population cohort[J]. *Nat Genet*, 2022, 54(2): 134–142.

[9] Lopera-Maya EA, Kurilshikov A, van der Graaf A, et al. Effect of host genetics on the gut microbiome in 7, 738 participants of the Dutch Microbiome Project[J]. *Nat Genet*, 2022, 54(2): 143–151.

[10] Ray D, Chatterjee N. A powerful method for pleiotropic analysis under composite null hypothesis identifies novel shared loci between Type 2 Diabetes and Prostate Cancer[J]. *PLoS Genet*, 2020, 16(12): e1009218.

[11] Hall JA, Pokrovskii M, Kroehling L, et al. Transcription factor RORα enforces stability of the Th17 cell effector program by binding to a Rorc cis-regulatory element[J]. *Immunity*, 2022, 55(11): 2027–2043.e9.

[12] Chi X, Jin W, Bai X, et al. RORα is critical for mTORC1 activity in T cell-mediated colitis[J]. *Cell Rep*, 2021, 36(11): 109682.

[13] Liu Z, Liu R, Gao H, et al. Genetic architecture of the inflammatory bowel diseases across East Asian and European ancestries[J]. *Nat Genet*, 2023, 55(5): 796–806.

[14] Li J, Bansal V, Tiwari M, et al. ELL facilitates RNA polymerase II-mediated transcription of human epidermal proliferation genes[J]. *J Invest Dermatol*, 2021, 141(5): 1352–1536.e3.

[15] Afrache H, Gouret P, Ainouche S, et al. The butyrophilin (BTN) gene family: From milk fat to the regulation of the immune response[J]. *Immunogenetics*, 2012, 64(11): 781–794.

[16] Abeler-Dörner L, Swamy M, Williams G, et al. Butyrophilins: An emerging family of immune regulators[J]. *Trends Immunol*, 2012, 33(1): 34–41.

[17] Rhodes DA, Chen HC, Price AJ, et al. Activation of human γδ T cells by cytosolic interactions of BTN3A1 with soluble phosphoantigens and the cytoskeletal adaptor periplakin[J]. *J Immunol*, 2015, 194(5): 2390–2398.

[18] Wu Z, Lamao Q, Gu M, et al. Unsynchronized butyrophilin molecules dictate cancer cell evasion of Vγ9Vδ2 T-cell killing[J]. *Cell Mol Immunol*, 2024, 21(4): 362–373.

[19] Zhang Q, Shi P, Wang Z, et al. Identification of the BTN3A3 gene as a molecule implicated in generalized pustular psoriasis in a Chinese population[J]. *J Invest Dermatol*, 2023, 143(8): 1439–1448.e21.

[20] Li Q, Chu Y, Yao Y, et al. A Treg-related riskscore model may improve the prognosis evaluation of colorectal cancer[J]. *J Gene Med*, 2024, 26(2): e3668.

[21] Baeyens AAL, Schwab SR. Finding a way out: S1P signaling and immune cell migration[J]. *Annu Rev Immunol*, 2020, 38: 759–784.

[22] Kitsou K, Kokkotis G, Rivera-Nieves J, et al. Targeting the sphingosine-1-phosphate pathway: New opportunities in inflammatory bowel disease management[J]. *Drugs*, 2024, 84(10): 1179–1197.

[23] Rosenbaum DM, Rasmussen SGF, Kobilka BK. The structure and function of G-protein-coupled receptors[J]. *Nature*, 2009, 459(7245): 356–363.

[24] Wootten D, Christopoulos A, Marti-Solano M, et al. Mechanisms of signalling and biased agonism in G protein-coupled receptors[J]. *Nat Rev Mol Cell Biol*, 2018, 19(10): 638–653.

[25] Sassone-Corsi P. The cyclic AMP pathway[J]. *Cold Spring Harb Perspect Biol*, 2012, 4(12): a011148.

[26] Coates M, Lee MJ, Norton D, et al. The skin and intestinal microbiota and their specific innate immune systems[J]. *Front Immunol*, 2019, 10: 2950.

[27] Trerotola M, Relli V, Simeone P, et al. Epigenetic inheritance and the missing heritability[J]. *Hum Genomics*, 2015, 9(1): 17.

[28] Allis CD, Jenuwein T. The molecular hallmarks of epigenetic control[J]. *Nat Rev Genet*, 2016, 17(8): 487–500.

[29] Porter DL, Levine BL, Kalos M, et al. Chimeric antigen receptor-modified T cells in chronic lymphoid leukemia[J]. *N Engl J Med*, 2011, 365(8): 725–733.

[30] Müller F, Taubmann J, Bucci L, et al. CD19 CAR T-cell therapy in autoimmune disease—a case series with follow-up[J]. *N Engl J Med*, 2024, 390(8): 687–700.

[31] Philips RL, Wang Y, Cheon H, et al. The JAK-STAT pathway at 30: Much learned, much more to do[J]. *Cell*, 2022, 185(21): 3857–3876.

[32] Shalabi MMK, Garcia B, Coleman K, et al. Janus kinase and tyrosine kinase inhibitors in dermatology: A review of their utilization, safety profile and future applications[J]. *Skin Therapy Lett*, 2022, 27(1): 4–9.

[33] Feagan BG, Rutgeerts P, Sands BE, et al. Vedolizumab as induction and maintenance therapy for ulcerative colitis[J]. *N Engl J Med*, 2013, 369(8): 699–710.

[34] Rotondi M, Chiovato L, Romagnani S, et al. Role of chemokines in endocrine autoimmune diseases[J]. *Endocr Rev*, 2007, 28(5): 492–520.

[35] Wu H, Ballantyne CM. Inflammation versus host defense in obesity[J]. *Cell Metab*, 2014, 20(5): 708–709.

[36] Cai Y, Xie S, Jia X, et al. Integrated analysis of Mendelian Randomization and Bayesian colocalization reveals bidirectional causal association between inflammatory bowel disease and psoriasis[J]. *Ann Med*, 2023, 55(2): 2281658.

[37] Shen S, Gao X, Song X, et al. Association between inflammatory bowel disease and rosacea: A bidirectional two-sample Mendelian randomization study[J]. *J Am Acad Dermatol*, 2024, 90(2): 401–403.

[38] Gu X, Ke X, Shen M, et al. Association between inflammatory bowel disease and atopic dermatitis: A two-

sample Mendelian randomization study[J]. *Inflamm Bowel Dis*, 2022, 28(2): e27–e28.

[39] Meisinger C, Freuer D. Causal association between atopic dermatitis and inflammatory bowel disease: A 2-sample bidirectional Mendelian randomization study[J]. *Inflamm Bowel Dis*, 2022, 28(10): 1543–1548.

[40] Chen Y, Wu C, Chen Y. Gastrointestinal comorbidities in patients with acne vulgaris: A population-based retrospective study[J]. *JAAD Int*, 2025, 18: 62–68.

[41] Teofani A, Marafini I, Laudisi F, et al. Intestinal taxa abundance and diversity in inflammatory bowel disease patients: An analysis including covariates and confounders[J]. *Nutrients*, 2022, 14(2): 260.

[42] Riazati N, Kable ME, Stephensen CB. Association of intestinal bacteria with immune activation in a cohort of healthy adults[J]. *Microbiol Spectr*, 2023, 11(6): e0102723.

[43] Gao Z, Chen K, Mueller O, et al. Microbiota of inflammatory bowel disease models[J]. *Annu Int Conf IEEE Eng Med Biol Soc*, 2018, 2018: 2374–2377.
